# Enhanced Light–Matter Interaction in Porous Silicon Microcavities Structurally Optimized Using Theoretical Simulation and Experimental Validation

**DOI:** 10.3390/nano15231808

**Published:** 2025-11-29

**Authors:** Evelyn Granizo, Irina S. Kriukova, Aleksandr A. Knysh, Pavel M. Sokolov, Pavel S. Samokhvalov, Igor R. Nabiev

**Affiliations:** 1 Research Center Nano-Photon, National Research Nuclear University MEPhI (Moscow Engineering Physics Institute), 115409 Moscow, Russia; aleroman16@hotmail.com (E.G.); i.krukova@lift.center (I.S.K.); knyshkikai@mail.ru (A.A.K.); socolovpm87@mail.ru (P.M.S.); p.samokhvalov@gmail.com (P.S.S.); 2 Life Improvement by Future Technologies (LIFT) Center, 121205 Moscow, Russia; 3 Department of Clinical Immunology and Allergology, Sechenov First Moscow State Medical University (Sechenov University), 119146 Moscow, Russia; 4 Université de Reims Champagne-Ardenne, BioSpecT UR-7504, 51100 Reims, France

**Keywords:** light–matter interaction, optical microcavities, porous silicon, electrochemical etching, quality factor, fluorescence

## Abstract

Light–matter interactions in optical microcavities attract much attention due to their potential for controlling properties of materials. Among the various types of optical microcavities, porous silicon microcavities (pSiMCs) are of special interest because of their relatively simple fabrication procedure, tunable porosity, and large specific surface area, which make them highly suitable for a wide range of optoelectronic and sensing applications. However, the fabrication of pSiMCs with precisely controlled parameters, which is crucial for effective light–matter coupling, remains challenging due to the multiple variables involved in the process. In addition, the parameter characterizing the capacity of pSiMCs for confining light inside the cavity (the quality factor, QF) rarely exceeds 100. Here, we present advanced methods and protocols for controlled fabrication of pSiMCs at room temperature, combining theoretical and numerical simulations and experimental validation of microcavity structural parameters for enhancing light–matter interactions. This systemic approach has been used to design and fabricate pSiMCs with an about twofold increased QF and correspondingly improved optical performance; the theoretical modeling shows that its further development is expected to increase the QF even more. In addition, we fabricated hybrid fluorescencent structures with the R6G dye embedded into the optimized pSiMCs. This provided a 5.8-fold narrowing of the R6G fluorescence spectrum caused by light–matter coupling, which indicated enhancement of the fluorescence signal at the eigenmode wavelength due to an increased rate of spontaneous emission in the cavity. The proposed methodology offers precise theoretical simulation of microcavities with the parameters required for specific practical applications, which facilitates optimization of microcavity design. The controllable optical properties of pSiMCs make them promising candidates for a wide range of applications where improved spectral resolution, and increased luminescence efficiency are required. This paves the way for further innovations in photonic systems and optoelectronic devices.

## 1. Introduction

Optical microcavities are a cornerstone of modern photonics, enabling unprecedented control over light–matter interactions through subwavelength confinement of light. This unique property makes them highly valuable for various applications, such as sensing [1], energy transfer [2], and a number of quantum-photonic applications that require single-photon sources [3]. Their key advantage is the possibility of substantially modifying the optical properties of materials integrated into the cavity, thus enabling ultrasensitive, e.g., single-molecule detection [4,5]. Recent advances in optical microcavity design and signal post-processing have significantly improved the scalability of this technology, facilitating the transition from laboratory research to complex real-world scenarios.

The most common types of optical microcavities are plasmonic, whispering-gallery mode (WGM), and Fabry–Pérot (FP) microcavities.

The microcavities containing plasmonic materials have been extensively explored due to the possibility of employing plasmonic effects that arise from the interaction of light with collective oscillations of free electrons [5,6,7]. Metal nanoparticles are often incorporated in microcavities of different types [8,9,10,11]. However, their use entails some challenges, such as the necessity of ensuring uniformity in the manufacturing process, which hinders the scalability of their production for practical applications.

WGM microcavities confine light through total internal reflection, ensuring minimal optical losses. Numerous types of WGM microcavities have been developed, differing in geometry [12,13,14,15,16,17] and material [18,19,20,21,22,23,24,25,26]. However, WGM microcavities also have some drawbacks in terms of practical applications, including their high sensitivity to environmental noise [27].

FP microcavities operate on the principle of multiple reflections between highly reflective mirrors, which can be either metal, dielectric, or semiconductor. They are fabricated by spacing two mirrors at a distance *L*, typically meeting the condition *L* = *m* × *λ*/(2*n*), where *m* is an integer, *λ* is the resonance wavelength, and *n* is the refractive index of the cavity medium. FP microcavities using simple mirrors, made of metal (e.g., gold, silver, or aluminum) [28,29,30,31], suffer from high optical losses. Distributed Bragg reflectors (DBRs) [32,33] are more sophisticated multilayer structures allowing minimization of the losses. DBRs are one-dimensional (1D) photonic crystals formed from periodic alternating layers with different refractive indices, each with an optical path of a quarter of the resonance wavelength, to maximize reflectivity via constructive interference [34]. These layers are made of different materials, including inorganic dielectrics (e.g., SiO_2_ and TiO_2_) [35], semiconductors (e.g., GaAs and AlGaAs) [36], and polymers [37].

One of the most convenient materials for DBRs is porous silicon (pSi), a material with unique optical and structural characteristics, including tunable pore size and porosity [38], discovered back in 1956 [39,40]. One of the earliest findings was that pSi can photoluminesce in the visible region at room temperature [41], a property that conventional crystalline silicon lacks.

PSi has a one to two orders of magnitude lower thermal conductivity compared with bulk silicon, this value depending on pSi porosity and surface passivation [42]. Due to its large specific surface area, pSi is highly sensitive to the environment. The properties of pSi can be modified by filling its pores with various materials. PSi is widely used as a host matrix for the immobilization of nanomaterials, e.g., quantum dots (QDs) [43], due to its tunable porosity. All these properties make pSi attractive for biosensing applications [44].

In biomedicine, pSi is favored for its biocompatibility [45,46]. However, freshly etched pSi is an unstable material, especially when exposed to air, moisture, or biological media. This instability is due to the large internal surface area and the presence of Si–H bonds on the pore surface, which are easily oxidized or hydrolyzed. Therefore, stabilization of pSi is important for practical applications. Several methods have been proposed to enhance its stability, including thermal oxidation [47], thermal carbonization [48], hydrosilylation [49] and atomic layer deposition [50].

The properties of pSi can be precisely tailored by carefully controlling the fabrication parameters, with the characteristics of the silicon substrate itself, such as its resistivity and crystallographic direction, taken into account. Numerous parameters, such as the thickness of the etched layer, porosity degree, and pore morphology and distribution, are interrelated and should be optimized during fabrication to obtain the desired properties. The International Union of Pure and Applied Chemistry (IUPAC) subdivides pSi according to pore size into microporous (pore diameter *d* < 2 nm), mesoporous (2 < *d* < 50 nm), and macroporous (*d* > 50 nm) [51]. The pores may be straight or branched, cylindrical or pyramidal. The morphology of the pores is also influenced by the electrolyte composition.

The most common solutions used in electrochemical etching contain fluorides [52]; in some cases, organic solvents are also added [53,54]. By modulating the current during the electrochemical etching process, it is possible to fabricate multilayer structures with varying porosity. This enables the production of advanced optical devices [55,56], including porous silicon microcavities (pSiMCs) [57].

The refractive index of pSi can be easily controlled by porosity during electrochemical etching, making it highly adaptable for various photonic applications. The refractive index of pSi is inversely related to its porosity: the higher the porosity of the layer, the lower its refractive index. Therefore, pSiMCs offer exceptional spectral tunability because their parameters can be precisely controlled during electrochemical etching.

PSiMCs have proved to be promising basic elements for sensors and tunable optical filters, making them valuable for applications in quantum technologies and photonics [57,58]. For example, β-lactoglobulin (β-LG) has been detected using pSiMCs with a detection limit of 0.73 μg/L using anti-β-LG antibodies conjugated to carbon QDs [59]. The reflection signal was recorded at different angles before and after the immune response. The reported experimental results show a linear relationship between the detection angle measured by reflection angle spectroscopy and β-LG antigen concentration. Gibberellins were also identified by analyzing fluorescence images of pSiMC recorded before and after the immunoreaction [60]. In this study, the gibberellin antibodies were conjugated with CdSe/ZnS QDs as a fluorescent marker. The detection limit for gibberellins was 20.05 pg/mL. Other studies have shown the possibility of using pSiMCs as optical chemosensors. Immersion of pSiMCs into different solvents changed their modes proportionally to the differences between the effective refractive indices of the solvents, which could be used as an analytical signal [61]. Embedding of CdSe QDs into pSiMCs resulted in a narrowed fluorescence spectrum and accelerated spontaneous emission rate of the QDs [43,62]. Other examples include the incorporation of perovskites within pSiMCs [63]. Advances in optical microcavity engineering, together with progress in the fabrication of perovskites with improved stability and luminous efficiency [64,65], would contribute to higher performance and enable new functionalities in integrated optical systems. A high potential of tunable optical filters and bistable laser resonators based on pSiMCs has also been demonstrated [66]. In this case, pSiMCs were filled with a nematic photochromic mixture based on an azobenzene derivative. By exposing this system to UV light, the photonic band gap is shifted by ~10 nm due to the *E*–*Z* isomerization of azobenzene. This process is fully reversible and can be activated by visible light and repeated many times without degradation of the photochromic mixture.

One of the most common and low-cost techniques for fabricating pSiMCs is electrochemical etching (anodization) of silicon, resulting in a material with morphological, optical, electronic, and mechanical properties different from bulk crystalline silicon. The morphology and architecture of pSiMC layers are influenced by various factors of the anodization procedure, including the etching solution concentration, current density, power supply mode (stationary or pulsed), bulk crystalline silicon resistivity, etching duration, and parameters of external energy supply, such as light exposure, temperature, and mechanical stress. As a result, the porous structure can be formed either as a randomly oriented, sponge-like network or as vertically aligned pores on the bulk substrate.

The continued development of pSiMCs has shown their importance for the advancement of photonic devices and their potential impact on a wide range of industries. However, despite the obvious progress in this field, challenges remain regarding the control of pSi morphology. Additionally, no standardized method is available for the mass production of pSiMCs, mainly due to the sensitivity of the initial silicon wafer resistivity to various fabrication conditions. Therefore, there is an ongoing need for the development of photonic devices that can be adapted to changing conditions in real time, as well as the development of fabrication methods that would meet specific requirements for the pSi structure.

One of the critical challenges in pSi research remains the control and reproducibility during its fabrication. In addition to common ex situ characterization techniques, such as gravimetry and cross-section scanning electron microscopy (SEM) for thickness measurement, in situ techniques have been proposed. For example, Fourier transform infrared (FTIR) spectrometry, optical interferometry, and photoacoustics have been used for monitoring several parameters during the electrochemical etching in hydrofluoric acid (HF). FTIR spectroscopy is used to identify surface chemical species and bonding structures, which significantly influence the optical properties of pSi. This technique makes it possible to identify characteristic groups, such as Si–H, Si–O_2_, and Si–OH, as well as potential surface functionalization [67]. FTIR spectroscopy can be used to evaluate porosity by measuring the concentration of Si–H, which is proportional to the surface and porosity. Optical interferometry is used to monitor the changes in the thickness, etching rate, refractive index, porosity, and interface roughness of porous silicon layers due to the constant changes in the optical path, resulting in constructive and destructive interference of light during the etching [68,69]. Photoacoustics is used not only to monitor the refractive index, etching rate, and porosity, but also the thermal properties of pSi [70,71]. The etching current amplitude is a periodic function of time. Unusual changes in the photoacoustic signal indicate a disturbance in the etching process in real time. Alternatively, when combined with other techniques, such as genetic algorithms and specular reflectance, the precise value of porosity can be determined without the need to estimate the thickness by means of microscopy. All these techniques allow precise real-time control of the etching process, improving the quality of pSi structures with the desired characteristics and allowing feedback during the manufacturing process.

Other strategies for optimizing the microcavity design include the use of evolutionary algorithms and artificial intelligence. Evolutionary algorithms are based on the iterative improvement in parameters by selecting and combining the best-performing conditions and take into account random changes called “mutations”. This method allows adjusting specific parameters, such as the thicknesses of DBR layers [72] and mirror configurations [73], maximizing the reflectance, determining the optimal number of layers, and increasing the mechanical stability of the porous silicon structures [74] so as to enhance light–matter interactions due to high-finesse cavity eigenmodes, large field enhancement at the center of the cavity, and the possibility to overcome limitations of traditional symmetric configurations. By using artificial intelligence methods, such as reinforcement learning, DBRs with structures other than traditional configurations can be designed. These methods promise better performance than conventional ones, such as TMM, and can be adapted to 3D configurations [75]. Additionally, artificial neural network models are used to suggest optimal design schemes [76,77] or predict broadband and modulated optical responses in one-dimensional pSi photonic crystals [78]. However, some potential challenges need to be evaluated, related to practical applicability and manufacturing issues.

These advances in optical microcavity design, including the potential benefits of disordered materials [79], have demonstrated the ability to achieve high-Q resonances under specific conditions. However, evolutionary algorithms are often time-consuming, sensitive to the initial values, or prone to convergence issues, while artificial intelligence requires large training databases containing information on thousands of device designs. Additionally, these methodologies typically rely on fabrication schemes that are not directly compatible with pSi etching. In the case of pSi systems, any optimization algorithm or neural-network-based design strategy has to take into account additional complications, because their fabrication depends not only on geometry, but also on environmental factors and electrolyte depletion during etching. These practical considerations should be incorporated into the training to ensure better results and practicable designs.

Thus, an urgent task is to precisely control the fabrication conditions so as to obtain pSiMCs with desirable characteristics, including layer thickness and refractive index contrast, which is essential for efficient light confinement inside the cavity and, hence, optimal performance of the device. The light confinement is quantified by the quality factor (QF) of the MC. Enhancing the QF requires high reflectivity of the DBRs ensured by strictly controlled layer thicknesses that vary periodically throughout the whole structure. The development of the procedure of DBR fabrication requires robust theoretical models that would determine optimal etching times and currents. These models provide calibration curves that serve as essential tools for estimating the etching parameters and layer porosities required for effective confinement of light at the desired wavelength. These methods, in combination with experimental calibrations, provide a straightforward and experimentally realistic way to improve the QF of pSiMCs while remaining compatible with standard electrochemical etching procedures, offering a practical alternative to optimization frameworks.

We have developed a procedure for fabricating high-performance pSiMCs by means of electrochemical etching under precisely controlled conditions. The procedure employs theoretical simulation for determining the pSiMC structure, selection of the material, optimization of the etching parameters, and accurate characterization of the fabricated structures. The theoretical simulation is combined with experimental validation for obtaining calibration curves that can guide the fabrication of uniform pSi monolayers and microcavities with the desired characteristics. Each pSiMC consists of two DBRs formed by alternating low- and high-porosity layers with a quarter-wave optical path separated by a double (half-wave) cavity layer. We estimated the effects of substrate resistivity and periodic arrangements of the layers on the morphology and optical properties of the resultant pSiMC. The effects of different fabrication parameters were estimated by analyzing the reflectance spectra and cross-sectional SEM imaging. Optimization based on these estimations has resulted in an about twofold increase in the QF of the microcavities and the corresponding enhancement of their optical performance. We have also demonstrated that embedding of the R6G dye into the pSiMCs results in a 5.8-fold narrowing of the emission spectrum caused by weak light–matter coupling in the hybrid structure. Thus, our systematic approach enables precise theoretical simulation and fabrication of pSi structures whose parameters can be customized for the requirements of various practical applications. The results of our study show the potential for designing novel photonic, optoelectronic, and biosensing hybrid devices.

## 2. Theoretical Models

Here, we have used theoretical models to determine the optimal parameters for designing optimized pSiMCs. The results of theoretical simulation guide the design of pSiMCs, optimize the etching procedure, and aid in better understanding the microcavity structure and predicting pSiMC performance before fabrication. For precise calculations using theoretical models and for calibration of the etching parameters and further optimization of the pSiMC structure, the approximation functions for refractive indices of Si and SiO_2_ are used. Detailed description of these approximation functions is presented in the Supporting Information, Section S1.

The calculations start with the Bruggeman effective medium approximation to estimate the effective refractive index of pSi layers that form the microcavity. In the case of pSi, the effective refractive index depends on the volume fractions of its three constituents: silicon (Si), air-filled pores, and silicon dioxide (SiO_2_) formed on the pore surface due to spontaneous oxidation of silicon [80]. Then, the layer thickness and the etching times are calculated using calibration curves. These parameters are used in the transfer matrix model to simulate light propagation through a layered medium and predict its optical response. This method is applicable only to 1D layered structures, where the refractive index varies in a single direction, and allows accurate calculation of the reflection and transmission spectra of multilayer structures [81,82], such as pSiMCs and DBRs. The results enable the design of optical components with desired optical properties. Detailed description of these methods is presented in the Supporting Information, Sections S2 and S3. An example of the calculation of the parameters and optical properties of a pSiMC using the Bruggeman approximation and the transfer matrix model (TMM) is also presented in Section S7 of the Supporting Information.

Alternatively, numerical methods, such as the finite element method (FEM) and the finite-difference time-domain (FDTD) method, could be used for more complex simulations, such as those of materials with strong absorption or scattering. the FEM and FDTD methods are numerical methods used for solving Maxwell’s equations in the analysis of electromagnetic wave propagation in complex two-dimensional (2D) and three-dimensional (3D) multimaterials [83,84]. These methods accurately simulate processes in strongly scattering, resonant, or highly absorptive materials, which makes them essential for studying pSiMCs. Both methods are based on the subdivision of the entire structure to be simulated and analyzed (the computational domain) into smaller elements to obtain an approximate solution. The FDTD method excels in understanding light–matter interactions extended in time, which allows the study of near and far fields, and the FEM enables the study of various photonic systems with intricate geometries. The methods are described in more detail in the Supporting Information, Section S4.

In summary, the Bruggeman effective medium approximation, analytical transfer matrix method, and numerical methods allow a better understanding of the relationships between the pSiMC structure and its optical properties. The TMM offers a rapid spectral analysis of ideal 1D layered systems, whereas FEM and FDTD simulations are used to overcome TMM limitations by solving Maxwell’s equations in the time and frequency domains.

## 3. Materials and Methods

### 3.1. Materials

Two types of boron-doped p^+^-type silicon wafers with the (100) crystallographic orientation were used: single-sided (S1) polished wafers (lapped on the back side) with a resistivity of 0.001–0.005 Ω·cm and double-sided (S2) polished wafers with a resistivity of 0.004–0.006 Ω·cm. Wafers of both types were purchased from Telecom-STV (Moscow, Russia).

Hydrofluoric acid (HF, 48%) was purchased from Component-Reaktiv (Moscow, Russia). Ethanol, methanol, isopropyl alcohol, hexane, polyvinylpyrrolidone (PVP, 10 kDa) and Rhodamine 6G (R6G) dye were purchased from Sigma-Aldrich (St. Louis, MO, USA) and used as received without purification.

### 3.2. Experimental Procedure

The fabrication of pSiMCs by electrochemical etching requires precise control of various conditions to achieve high QFs of the resultant cavities. Key aspects include selecting a material that allows obtaining a high refractive index contrast, ensuring a clean substrate surface and etching cell walls, implementing thermal stabilization, and optimizing electrical contacts with the anode and cathode. Furthermore, careful control of the etching current (e.g., by using a programmable power source), optimization of etching parameters, and thorough post-etching cleaning (e.g., by rinsing in an appropriate solvent) are essential for obtaining high-QF microcavities.

We used boron-doped p-type silicon wafers with the (100) crystallographic orientation. The wafers had a thickness of approximately 380 µm and were used to fabricate microcavities with depths of about 5 µm and a surface diameter of about 1 cm. The original wafer was carefully cut into small pieces, each about 1.5 cm long and wide. Before etching, the surface of the silicon substrates was cleaned as follows. First, the substrates were immersed in methanol for 5 min; then, the solvent was decanted, and the substrates were ultrasonicated in isopropyl alcohol for 15 min and cleaned again in methanol for 5 min in order to remove the surface impurities. All the substrates were stored in methanol in a sealed tube at room temperature until etching. Immediately before etching, the substrate was placed into a mixture of hydrofluoric acid (HF solution) and ethanol at a volume ratio of 1:7 for about 5 min, and then it was left to dry in the air.

**Figure 1:**
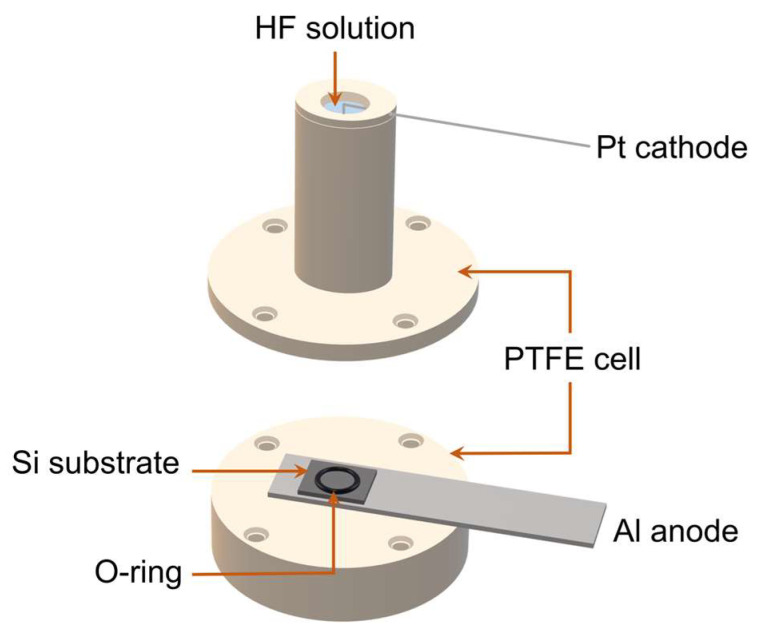
Schematic representation of the electrochemical etching setup.

Electrochemical etching was performed in an etching cell made of polytetrafluoroethylene (PTFE), which is inert to hydrofluoric acid (Figure 1). The setup included an aluminum (Al) foil as a replaceable anode contact and a platinum (Pt) wire as a cathode. Once the silicon substrate was cleaned, it was placed on top of the Al foil so that only the polished side of the silicon substrate contacted the etching solution. An O-ring made of acid-resistant rubber was used as a sealer between the Si substrate and the top part of the PTFE etching cell. The substrate was etched by electrochemical anodization at room temperature in a mixture of HF and ethanol at a volume ratio of 3:7. During the etching of individual pSi layers, a constant current from a 2635A programmable power source (Keithley Instruments, Solon, OH, USA) was applied to the cell for a specified period of time. When the etching was finished, the solution was removed and the setup was immediately cleaned with ethanol. Afterwards, the substrate was rinsed and shaken in methanol for approximately 2 min and afterwards in hexane for 2 min in order to remove residual etching solution from the pores and to minimize the risk of pSi cracking because of the strong capillary forces and thermal stresses arising as ethanol evaporated from the pores.

Before pSiMCs with predictable optical properties could be fabricated, we performed preliminary experiments on the calibration of the layer porosity and etching rate as functions of the etching current. This is a necessary step with every new batch of Si wafers, even with a known electrical resistivity. For calibration, pSi monolayers, i.e., individual layers with a fixed porosity that did not change during the etching process, were first fabricated under a constant current and characterized using SEM and gravimetry. Thus, the empirical ratios between the etching conditions (etching current and time) and the characteristics of the resultant material (porosity and thickness) were established. These calibration curves served as a basis for further precise control of the fabrication of the multilayer structures.

**Figure 2:**
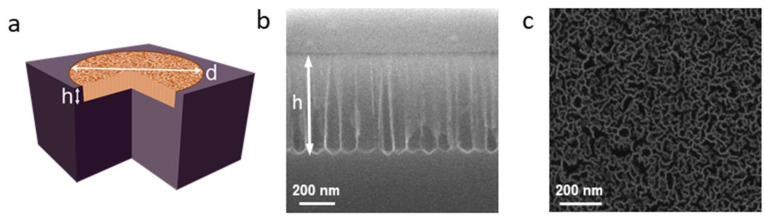
(**a**) Schematic representation of a porous silicon monolayer; d and h are the diameter and thickness of the etched area, respectively. (**b**) An SEM image of a cross-section of the porous silicon monolayer. (**c**) An SEM image of the porous silicon monolayer surface.

In the calibration experiments, we performed the etching procedure using a series of substrates made from a single Si wafer (~8 cm in diameter), with the necessary amount of the 3:7 HF–ethanol mixture prepared in advance for all samples to minimize the effects of the possible variations in the silicon resistivity and the etching-electrolyte composition. In addition, all the monolayers required for calibration are prepared on the same day to minimize any potential variations in the external parameters, such as air temperature and humidity. A modified gravimetric method was used to determine the porosity of the monolayers. The monolayer samples were weighed before and immediately after etching and washing; then, their porosity can be calculated as the mean volume fraction of Si removed from the sample:

(1) $$P=\frac{m_{1}-m_{2}}{V\cdot \rho_{Si}}\cdot 100\%,$$

(2) $$V=\frac{\pi d^{2}}{4}\cdot h,$$

where *m*_1_ and *m*_2_ are the weights of the silicon wafer before and after etching, *ρ_Si_* is the density of silicon; *V*, *d*, and *h* are the volume, diameter, and thickness of the etched area, respectively. Figure 2a schematically shows the monolayer sample. The thicknesses of the etched layers were estimated from cross-sectional SEM images. Figure 2b,c show SEM images of the cross-section and surface of a monolayer sample obtained from an S1 polished silicon wafer with a resistivity of 0.001–0.005 Ω·cm. The SEM images of monolayers fabricated at different etching parameters are shown in Supporting Information, Section S5. The characteristic pore width ranges from approximately 10 to 65 nm for low- and high-porosity layers, respectively. Surface images reveal a sponge-like pore arrangement, while cross-sectional images show that the pore walls in low-porosity layers are irregular, with increasing etching current leading to smoother pore walls.

**Figure 3:**
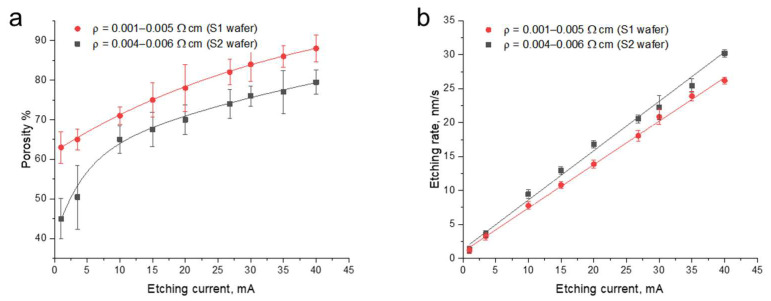
The (**a**) porosity and (**b**) etching rate as functions of the current used for the electrochemical etching of p^+^-type boron-doped silicon substrates with the (100) orientation and with resistivities of 0.001–0.005 and 0.004–0.006 Ω·cm for single-sided (S1) and double-sided (S2) wafers, respectively. The etching solution was a mixture of 48% HF and ethanol at a volume ratio of 3:7. The dots and lines in (**a**) and (**b**) represent the experimental values and approximation functions used in the TMM, respectively.

However, we found that the calibration results could be distorted not only due to the error of weight measurements, but also because the porosity was lower at the edges of the etched area than in its center. Therefore, in addition to the gravimetric method, we estimated the porosity (Figure 3a) by comparing the experimental reflectance spectra of pSi monolayers with those derived from the TMM, with the thickness of the pSi layer estimated from SEM images.

In addition, monolayers with the same expected layer thickness were prepared in order to obtain the calibration curve for the etching rate, i.e., the speed at which the pores are formed by removing silicon during the electrochemical etching. We used the time of etching, either roughly estimated from the calibration curves that we constructed earlier for substrates with a similar resistivity or taken from published data. Once the etching time for each current was determined, these values were input into the etching software, and the etching of monolayers was carried out. After that, the real thicknesses of the pSi layers were measured using SEM. Then, with this experimental etching time (*t*) and monolayer depth (*d*), we determined the actual etching rate as *v = d/t* (Figure 3b).

Then, the approximation functions for the experimental data on the porosity and etching rate for the given etching conditions were input into the TMM to simulate and design pSiMCs with a multilayer structure.

The parameters obtained from TMM were used in fabricating pSiMCs whose top and bottom DBR were formed by 5 and 20 pairs of layers, respectively. The large number of layer pairs in the bottom DBR ensured a high QF of the microcavity and, when a luminophore was placed into the cavity, determined the direction of fluorescence signal propagation. On the other hand, the small number of layer pairs in the top DBR ensured its high transmittance and effective excitation of the pSiMC eigenmode. Alternatively, microcavities with six and ten pairs of layers in the top and bottom DBR, respectively, were fabricated. The layer thicknesses, as mentioned above, were always selected in such a way that the optical path through each layer was equal to a quarter of the projected wavelength of the pSiMC eigenmode in order to maximize the reflectivity of the DBRs. After etching, the pSiMCs were subjected to oxidation, which passivated the surface states and, hence, stabilized the pSiMC optical properties through the formation of a silicon oxide layer on the walls of the porous structure. In addition, oxidation helped suppress nonradiative relaxation of the excited luminophores embedded in the porous structure via Förster resonance energy transfer (FRET) [85] or direct charge carrier transfer [86]. Thermal oxidation was performed in ambient atmosphere at 600 °C for 2 h using a muffle furnace. A video of the entire procedure for the fabrication of pSi samples is presented in the Supporting Information. Additionally, the troubleshooting and safety considerations are presented in Section S6 of Supporting Information.

### 3.3. Characterization

Surface and cross-sectional images of the pSi structures were obtained using a MAIA3 Tescan scanning electron microscope (SEM) (Oxford Instruments, Abingdon, UK) at an accelerating voltage of 30.0 kV. The thicknesses of monolayers and layer thicknesses in microcavities were estimated from SEM images by means of the Fiji software (ImageJ 1.54p) [87]. In constructing porosity calibration curves by the gravimetric method, the weight of the substrates was measured using a CPA2P analytical balance (Sartorius, Göttingen, Germany) with an accuracy of 0.001 mg. Thermal oxidation of pSi samples was performed in a Nabertherm B180/1300 muffle furnace (Nabertherm, Lilienthal, Germany).

Reflectance and fluorescence spectra were measured using an USB2000+ spectrometer (Ocean Optics, Winter Park, FL, USA) with an SS diaphragm. The use of the diaphragm was essential for evaluating the optical properties of the fabricated microcavities. The fluorescence of the R6G solution was measured using a Cary Eclipse fluorescence spectrophotometer (Agilent Technologies, Santa Clara, CA, USA). The absorbance of R6G was measured using a Cary 60 UV-Vis spectrophotometer (Agilent Technologies).

## 4. Results and Discussion

### 4.1. Optimization of the Etching Time

We have developed an approach to the optimization of the electrochemical etching parameters in order to obtain pSiMCs with improved optical properties, in particular, an increased QF. The calibration curves obtained during etching of pSi monolayers showed an increase in the porosity and a decrease in the refractive index with increasing etching current. This was accompanied by an increase in the etching rate. These calibration curves were used for determining the optimal layer thickness (corresponding to a quarter-wavelength optical path) and predicting the optical properties of the multilayer microcavities, which made it possible to precisely control the fabrication process.

It should be noted that there are small but distinct differences between the etching processes in Si substrates with resistivities in the ranges of 0.001–0.005 Ω·cm (S1) and 0.004–0.006 Ω·cm (S2), even though these ranges are close to each other. Substrates with lower resistivities are more heavily doped, which results in a higher electrical conductivity and, hence, a faster etching of silicon in the lateral direction at a given etching current. As a consequence, these substrates are more susceptible to aggressive etching, which may increase the risk of the transition from the etching mode to the electropolishing one, especially for deeper and more porous layers, where the HF concentration is depleted. Electropolishing is the reaction mode in which the material is removed uniformly, without the formation of a porous structure, and the previously etched pSi layers are delaminated from the substrate. Etching from the surface into the depth of the material is slightly more rapid in substrates with higher resistivities at the same etching current, while etching in the lateral direction is slower. This results in smaller pore sizes, which is confirmed by SEM images. This makes it possible to use higher limiting currents for S2 wafers than for S1 ones without reaching the electropolishing regime, which ensures greater control over pore size and allows obtaining a higher porosity while avoiding delamination. In S1 substrates, the pores tend to merge more easily; therefore, structures with larger pores compared to S2 substrates are formed at the same etching current.

First, pSiMCs were fabricated with the top and bottom DBRs consisting of 5 and 20 pairs of alternating layers, respectively. The low- and high-porosity layers were formed under alternating low (3.5 mA) and high (26.8 mA) etching currents, which were determined from the calibration curves. The cavity between the DBRs was a double-thickness layer with low porosity. Below, we refer to these samples of pSiMCs as non-optimized ones.

**Figure 4:**
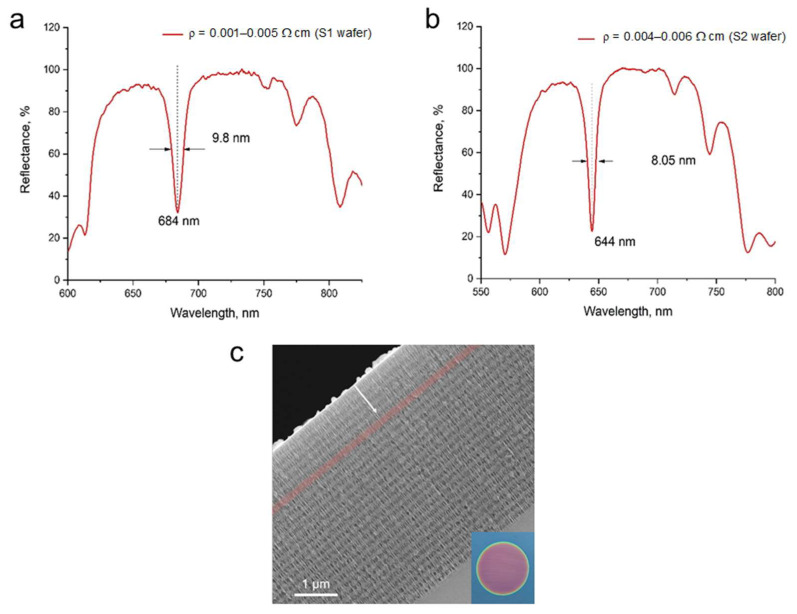
Reflectance spectrum of non-optimized pSiMCs made from boron-doped p^+^-type Si wafers with resistivities of (**a**) 0.001–0.005 Ω·cm (S1) and (**b**) 0.004–0.006 Ω·cm (S2). (**c**) A cross-section SEM image of a non-optimized pSiMC with the top and bottom DBRs consisting of 5 and 20 pairs of alternating layers, respectively. The arrow shows the direction of etching, the cavity is highlighted in red. The inset shows a photograph of the sample.

The reflection spectra of the non-optimized pSiMCs obtained using S1 and S2 wafers are shown in Figure 4a and Figure 4b, respectively. In the case of the pSiMCs made from S1 wafers, the reflection spectrum had an eigenmode at 684 nm with a FWHM of 9.8 nm, which corresponds to a QF of 69.8. A FWHM of the photonic band gap (i.e., the spectral range over which light transmission is prohibited due to Bragg reflection in the periodic layers of the pSiMC) was 184 nm. The pSiMCs produced from S2 wafers exhibited better confinement, with an eigenmode at 644 nm, a FWHM of 8.1 nm (QF ≈ 80), and a photonic band gap FWHM of 183 nm. Figure 4c shows a cross-sectional SEM image of a non-optimized pSiMC, where the multilayer architecture and a clearly distinguishable cavity between the DBRs (highlighted in red) can be seen. The inset shows a photograph of the sample (see Section S5 of Supporting Information for more photographs of the samples, together with surface and cross-sectional SEM images of pSi monolayers).

**Figure 5:**
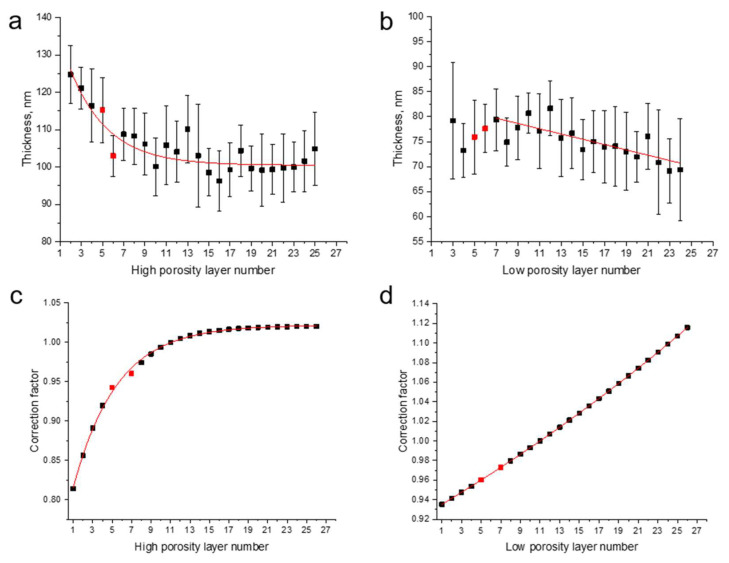
Variation in layer thickness in (**a**) high- and (**b**) low-porosity layers of porous silicon microcavities (pSiMCs) made from a substrate with a resistivity of 0.001–0.005 Ω·cm (S1) and the etching time correction factors for the (**c**) high- and (**d**) low-porosity layers. The black bars indicate the standard deviation of the layer thickness measurements. The red lines show the approximation functions for (**a**,**b**) thickness variations and (**c**,**d**) the time correction factors used for optimizing electrochemical etching of S1 wafers. The black dots indicate the layers in the top and bottom DBRs, and the red dots indicate the layers adjacent to the pSiMC cavity layer.

Analysis of cross-sectional SEM images of the pSiMCs described above revealed a systematic, gradual decrease in layer thickness from the sample surface towards the substrate (the white arrow in Figure 4c). This effect is attributed to (1) HF depletion, which slowed the etching process, and (2) the accumulation of hydrogen bubbles, which partly blocked the pores, causing localized etching inhibition. As shown in Figure 5a,b, the layers of the pSiMCs made from the S1 wafer exhibited a pronounced variation in thickness: 25 ± 3 nm for high-porosity layers and 9 ± 3 nm for low-porosity ones. The same trend was observed for pSiMCs made from S2 wafers, but the variation in layer thickness in them was considerably smaller: 10 ± 2 and 6 ± 1 nm for high- and low-porosity layers, respectively.

These variations in layer thickness can be attributed to diffusion-limited electrochemical etching kinetics. At high reaction rates, HF is consumed rapidly, and the local HF concentration is decreased. This results in deficiency of fluoride ions at the pSi–monocrystalline silicon interface, causing (1) a local porosity increase, (2) a reduction in etching rates in deeper layers, and (3) transition to the electropolishing mode at critical current densities [88]. This effect is more pronounced in the high-porosity layers of both substrates, S1 and S2, which are thicker than those of low porosity, because the high conductivity of the substrates ensures efficient carrier transport even at a low etching current (3.5 mA). However, in deeper layers, electrolyte is depleted, which causes slight thickness variations. At a high etching current (26.8 mA), the etching is faster, which leads to higher porosity of the layers, but also exacerbates the problem of electrolyte depletion and hydrogen bubble accumulation. As a result, a gradual decrease in the etching rate or even transition to the electropolishing mode at critical currents may occur in deeper layers, which compromises structural integrity. In the case of S2 wafers, their etching behavior allows the formation of more homogeneous layers throughout the structure. Therefore, the fabrication of pSiMCs from S2 wafers reduces the thickness gradient in their structure, which becomes closer to the ideal, strictly periodic one. In contrast, the more aggressive etching and a random lateral merging of pores along the layer, including the interface between the layers, in S1 wafers result in irregular layer interfaces with slightly different thicknesses along the layer structure. These inhomogeneities directly affect the optical properties of the microcavity along its surface.

**Figure 6:**
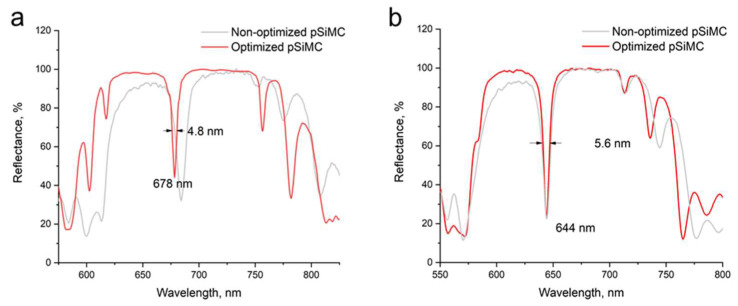
Experimental reflectance spectra of optimized (red line) and non-optimized (gray line) porous silicon microcavities (pSiMCs) made from wafers with resistivities of (**a**) 0.001–0.005 Ω·cm (S1) and (**b**) 0.004–0.006 Ω·cm (S2).

Analysis of cross-sectional SEM images of pSiMCs fabricated from a substrate with a resistivity of 0.001–0.005 Ω·cm showed the patterns of thickness changes in the high- and low-porosity layers with increasing etching depth (Figure 5a and Figure 5b, respectively). These data were used to make the appropriate corrections of the etching time for each individual layer and improve the periodicity of the pSiMC structure. The etching times were optimized to equalize the layer thicknesses throughout the structure. This means that shorter etching times were used for the upper layers, which were thicker, and longer etching times, for deeper layers. The red curves in Figure 5a,b are the approximations of the layer thickness variation used to calculate the correction factors for the etching times shown in Figure 5c,d. The approximation results and calculations for the pSiMCs made from S2 wafers were similar to those for pSiMCs from S1 wafers. In addition, in order to mitigate silicon passivation caused by hydrogen accumulation, we stopped the etching for 5 s after finishing each layer, which facilitated the dissipation of H_2_ bubbles. This prevented the interference of surface passivation with the etching process and made and the substrate accessible for the reaction with HF. The reflectance spectra of the pSiMCs fabricated using this optimized technique demonstrated significant improvement in their optical properties (Figure 6). The optimized pSiMCs made from S1 wafers had an eigenmode at 678 nm with an FWHM of 4.8 nm and a QF of 141.3 (Figure 6a); those made from S2 wafers had an eigenmode at 644 nm with an FWHM of 5.6 nm and a QF of 115.6 (Figure 6b). The photonic band gaps of both types of optimized pSiMCs were close to theoretically predicted ones, which indicated improved spectral restriction. SEM analysis confirmed that the optimized layers had approximately equal thicknesses throughout the pSiMC, which was crucial for good optical performance. Table 1 summarizes the main characteristics of the pSiMCs obtained using the optimized etching procedure. This method is also applicable in the NIR, in which case the layers should be thicker to meet the quarter-wave condition. Because the effective refractive indices of the pSi layers are wavelength-dependent, theoretical simulations should use other initial refractive indices for this spectral range. Then, SEM measurements can be used to calibrate etching times and homogenize layers. Moreover, absorption losses in the NIR are generally lower, which increases the QF.

### 4.2. Optimization of the Refractive Index Contrast and Layer Arrangement

**Figure 7:**
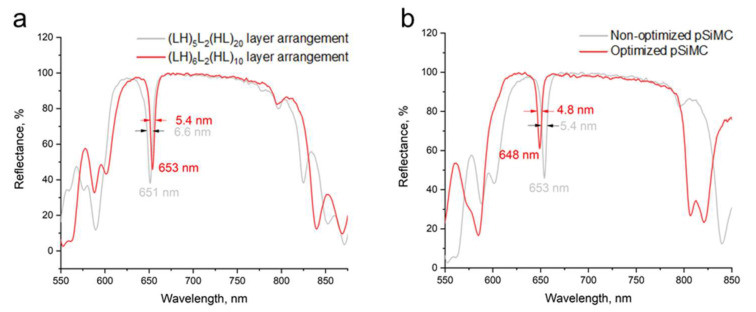
Experimental reflectance spectra of porous silicon microcavities (pSiMCs) with an increased refractive index contrast made from S2 wafers with a resistivity of 0.004–0.006 Ω·cm using etching currents of 40 and 1 mA. (**a**) Comparison of non-optimized pSiMCs (fabricated without using breaks or etching time gradient) with different layer structures: 5- and 20-layer pairs in the top and bottom distributed Bragg reflectors (DBRs), respectively ((LH)_5_L_2_(HL)_20_, gray) and 6- and 10-layer pairs in the top and bottom DBRs ((LH)_6_L_2_(HL)_10_, red). (**b**) Comparison of non-optimized and optimized (LH)_6_L_2_(HL)_10_ pSiMCs with an increased refractive index contrast.

Having reduced the variation in pSi layer thickness, we further improved the optical quality of pSiMCs by increasing the refractive index contrast between the layers comprising the cavity. We used S2 wafers, which exhibited smaller variation in the thickness. For this purpose, we fabricated pSiMCs with the same (LH)_5_L_2_(HL)_20_ configuration, but we used the etching currents of 1 mA and 40 mA, which were lower and higher than the low and high currents, respectively, used in the experiments described above. This was found to improve the optical characteristics of the pSiMCs made from S2 wafers: they had a distinct cavity eigenmode at 651 nm with an FWHM of 6.6 nm and a QF of 98.5. By using S2 wafers characterized by a higher resistivity, we prevented the electropolishing mode at high etching current, and, hence, preserved the integrity of the sample. Figure 7a (gray curve) shows the reflectance spectrum of non-optimized pSiMCs (fabricated without using etching time gradient or a break after etching each layer) with an increased contrast between the refractive indices of adjacent layers.

We also studied the effect of the number of layers in DBRs forming the microcavity on its performance. A series of simulations followed by experimental validation showed the pSiMCs with six and ten pairs of layers in the top and bottom DBRs, respectively ((LH)_6_L_2_(HL)_10_), to perform better than the (LH)_5_L_2_(HL)_20_ pSiMCs. More precisely, this configuration displayed three key advantages: (1) the fabrication time reduced by 30% compared to the (LH)_5_L_2_(HL)_20_ configuration, which is advantageous in terms of the optimal production routine; (2) a better optical performance, with an eigenmode at 653 nm, FWMH of 5.4 nm, and QF of 121.1 for non-optimized cavities; (3) a better spectrum shape, with a photonic band gap closer to the theoretically predicted one (Figure 7a).

The (LH)_6_L_2_(HL)_10_ pSiMCs fabricated using the optimized procedure had an eigenmode at 648 nm with an 11% narrower FWHM (4.8 nm) and, correspondingly, a higher QF (135.6) (Figure 7b). Table 2 summarizes the performance parameters, which demonstrate the effectiveness of our combined approach to the optimization of the fabrication of pSiMCs by electrochemical etching, including the 5 s breaks between the formation of individual layers and the use of the etching time gradient, with etching times corrected according to the results of SEM analysis, as well as the optimal numbers of layer pairs in the DBRs.

### 4.3. Embedment of a Fluorescent Dye into Optimized Porous Silicon Microcavities

Experiments with rhodamine 6G (R6G), a common fluorescent dye, embedded into the cavity layer have demonstrated that an optimized pSiMC can serve as an active optical component. We used this highly fluorescent dye because it frequently serves as a gain medium in dye lasers and is characterized by an extremely high photostability, a high fluorescence quantum yield (~95% in ethanol solution), and low cost [89]. R6G has a fluorescence maximum at 568 nm with a FWHM of about 35 nm. The pSiMCs fabricated from S2 wafers using the optimized procedure were thermally oxidized in order to stabilize the optical properties and suppress channels of nonradiative relaxation of excitation [90,91]. During the oxidation process, part of the silicon is transformed into silicon oxide, whose refractive index is lower than that of silicon (1.45 versus 3.5). This transformation results in a blue shift (also referred to as a hypsochromic shift) of the reflection spectrum by ~54 nm. This spectral shift indicates an oxidation level of approximately 70%, as estimated using the Bruggeman effective medium model for a three-component system consisting of silicon, silicon dioxide, and air [92]. As a result of oxidation, the contrast of the refractive indices of the pSiMC layers was decreased, which led to a 1.4-fold increase in the FWHM of the eigenmode and an 8% decrease in the photonic band gap (by ~14 nm). These systemic changes demonstrate the critical balance between stabilization of the optical properties and compromised performance of photonic structures made of oxidized porous silicon.

For experiments with embedded R6G, the optimized pSiMCs were carefully selected so that the wavelength of the cavity eigenmode matches that of R6G fluorescence, in order to maximize the light–matter interaction in the hybrid structure. A 0.07 mg/mL solution of R6G in ethanol was used for the experiment. Aggregation of R6G molecules on the walls of the porous structure was prevented by adding 8.1 mg of polyvinylpyrrolidone (PVP) with an average molecular weight of 10 kDa to 1.5 mL of the R6G solution. The resulting dye–polymer solution was embedded into the pSiMCs under a pressure of 3 bar. To do this, the pSiMCs were placed on the bottom of a 40 mL crystallization dish, and the R6G solution was poured into the dish in such a way that the meniscus of the solution was slightly above the top of the pSiMC substrate. The crystallization dish was placed into a sealed container pressurized to 3 bar using an air compressor.

**Figure 8:**
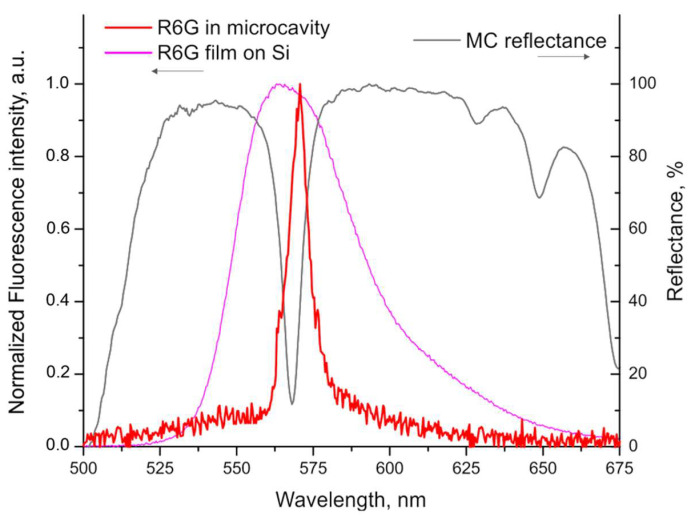
Fluorescence spectra of a rhodamine 6G film on a silicon substrate (magenta) and in a porous silicon microcavity (red). The black curve is the reflection spectrum of the porous silicon microcavity.

The embedding of R6G into the pSiMCs resulted in a pronounced narrowing of the fluorescence spectrum compared to both the R6G solution in ethanol and dry dye on the silicon surface. As seen in Figure 8, R6G embedded in pSiMC had a 5.8-fold narrower fluorescence linewidth than the R6G film on silicon (with an FWHM of 7.8 nm versus 45 nm), the emission peak perfectly matching the microcavity eigenmode. This behavior is characteristic of the Purcell effect, which is a marker of weak light–matter coupling between the pSiMC eigenmode and dye excitons in the hybrid system. The Purcell effect implies an enhanced spontaneous emission rate in the cavity compared to free space, because weak light–matter interaction modifies the local density of photonic states. Thus, comparison of the fluorescence spectra clearly shows that the microcavity can tailor molecular emission properties of the dye without altering its chemical structure.

The rate of spontaneous emission depends on the interaction between the emitter and its electromagnetic environment. Hence, changing this environment may modify spontaneous emission. When R6G is placed into a pSiMC under resonance conditions, the local density of optical states is increased at the location of the emitter because the electromagnetic field is strongly confined, and the photon lifetime in the cavity is increased, which increases the spontaneous emission rate. This effect can be quantified by the Purcell factor [93]:

(3) $$F_{P}=\frac{\Gamma_{cav}}{\Gamma_{0}}=\frac{3Q\lambda^{3}}{4{\pi^{2}n^{3}V}_{eff}},$$

where $\Gamma_{0}$ is the spontaneous emission rate for an emitter in free space; $\Gamma_{cav}$ is the spontaneous emission rate for an emitter in the cavity and is proportional to the local density of optical states at the location of the emitter and to the emission wavelength; *λ* is the emission wavelength of the dye; *n* is the refractive index of the cavity; *Q* is the QF of the cavity mode; and *V_eff_* is the effective mode volume. This means that a high QF combined with a small mode volume of the pSiMC increases the local density of optical states at the resonance wavelength. As a result, the spontaneous emission rate of R6G is enhanced, generating stronger emission intensity and spectral narrowing compared to free-space emission.

## 5. Conclusions

The QF depends on the type of resonator, as well as the material used for its fabrication, with a typical value of about 100 for pSiMCs [94,95,96,97]. It has been theoretically demonstrated that the QF of pSi cavities cannot exceed 180 in the visible range [98]. Here, we have described the experimental design and fabrication of pSi resonators with a QF that reaches 140, i.e., a value close to the theoretical limit, if the fabrication parameters are optimized. These QF values allow pSi cavities to be used for modifying the spectral and temporal characteristics of the fluorescence of embedded luminophores [99,100,101,102]. It is worth noting that cavities of this type can be fabricated at room temperature with the use of standard hydrofluoric acid solutions, which makes the fabrication simple and inexpensive. However, some changes in the fabrication conditions, e.g., a decrease in temperature to about −22.5 °C and stable maintaining it at this level, as well as a switch to the infrared range, make it possible to achieve an order of magnitude higher QF, i.e., 1500 [103]. This would enable the use of pSi cavities for engineering high-quality interferometric structures and highly selective wavelength filters [103,104]. At the same time, there are other types of cavities whose QFs vary in a wide range. For example, QFs of about 20 are typical of plasmonic nanocavities [105]. Despite the high loss rates, they exhibit subwavelength mode volumes and can significantly amplify the electric field near the hot spots. These nanocavities can be used for nanoscale label sensing and ultrasensitive single-molecule spectroscopy [105,106]. On the other hand, WGM cavities used for highly sensitive detection, including single-molecule ones, and in applications employing strong light–matter coupling [106,107,108], typically have substantially larger QFs, sometimes of the order of 10^8^ [109]. However, the fabrication of these advanced microcavities requires the use of significantly more expensive and sophisticated techniques. Therefore, despite the significant difference in the experimental values of QF of different types of cavities, there are relevant practical applications for each of them, with their own optimal QF values.

Here, we have developed a comprehensive methodology for the design, engineering, and fabrication of pSiMCs by means of controlled electrochemical etching at room temperature that makes it possible to optimize their structural parameters for enhancing light–matter interactions. This methodology integrates theoretical calculations based on the Bruggeman model and TMM, numerical simulations (using FDTD and FEM), experimental validation, and strategies for optimizing the process. The optimization includes a precise control of layer thickness, adjustment of the refractive index contrast and arrangement of the layers, and restoration of the local concentration of fluoride ions in the electrolyte by stopping the etching after the formation of each layer. This approach allows the pSiMC eigenmode linewidths to be decreased to less than 5 nm and its QF to be increased by a factor of two. The optimized pSiMCs efficiently modify the radiative properties of embedded emitters. In particular, the fluorescence spectrum of R6G dye embedded into the cavity can be narrowed 5.8-fold. This can be explained by the Purcell effect in the weak light–matter coupling regime, the spontaneous emission rate being increased because of the change in local density of photonic states when the emission frequency of the fluorophore is resonant with the cavity mode. Overall, the proposed methodology provides precise control over the fabrication of pSiMCs and allows obtaining high-quality cavities with tunable optical properties, thereby extending their potential use as versatile platforms for both fundamental studies on light–matter interaction and practical applications. The improvements in their structure and optical properties make pSiMCs promising candidates for the development of more performant photonic systems and optoelectronic devices.

Thus, the fabrication of high-quality pSiMCs is called for in practical applications employing light–matter interaction. In addition to ex situ and in situ techniques for controlling the etching process and parameters, approaches such as evolutionary algorithms and artificial intelligence have been recently introduced. However, challenges related to practical applicability and manufacturability still need to be addressed. In the specific case of pSiMCs, algorithms of the optimization or neural-network-based design strategies should take into account additional complications. Their fabrication depends not only on geometric parameters but also on other factors, such as environmental conditions, electrolyte depletion during etching, and substrate properties. Therefore, integrating these practical considerations summarized in our study into the training of artificial intelligence systems will definitely contribute to practicable high-performance designs of pSiMCs.

## Figures and Tables

**Table 1:** Comparison of the optical properties of (LH)_5_L_2_(HL)_20_ porous silicon microcavities fabricated using optimized and non-optimized techniques.

Microcavity Fabrication Technique	Wafer Type	Eigenmode Wavelength, nm	Eigenmode FWHM, nm	QF
Non-optimized	0.001–0.005 Ω·cm (S1)	684	9.8	69.8
	0.004–0.006 Ω·cm (S2)	644	8.05	80
Optimized	0.001–0.005 Ω·cm (S1)	678	4.8	141.3
	0.004–0.006 Ω·cm (S2)	644	5.6	115.6

**Table 2:** Comparison of the optical properties of porous silicon microcavities made from S2 wafer (0.004–0.006 Ω·cm) using optimized and non-optimized routines with different numbers of layers in the distributed Bragg reflectors.

Microcavity Fabrication Technique	Structure	Eigenmode Wavelength, nm	Eigenmode FWHM, nm	QF
Non-optimized	(LH)_5_L_2_(HL)_20_	651	6.6	95.5
	(LH)_6_L_2_(HL)_10_	653	5.4	121.1
Optimized	(LH)_6_L_2_(HL)_10_	648	4.8	135.6

## Data Availability

The original contributions presented in the study are included in the article/Supplementary Materials; further inquiries can be directed to the corresponding author.

## Supplementary Materials

The following Supporting Information can be downloaded at https://www.mdpi.com/article/10.3390/nano15231808/s1, (Section S1) approximation functions for refractive indices of Si and SiO_2_; (Section S2) the Bruggeman effective medium approximation; (Section S3) the transfer matrix model; (Section S4) numerical methods; (Section S5) SEM images of porous silicon monolayers and a photograph of porous silicon microcavities; (Section S6) troubleshooting and safety considerations (PDF); (Section S7) an example of calculation of the parameters and optical properties of a porous silicon microcavity using the transfer matrix model and Bruggeman approximation (PDF); a video of the entire process of fabrication of porous silicon samples (mp4). Refs. [110,111,112,113,114,115,116,117] are cited in the supplementary materials.

## Abbreviations

The following abbreviations are used in this manuscript:

CaF_2_	Calcium fluoride
DBRs	Distributed Bragg reflectors
FEM	Finite element method
FDTD	Finite-difference time-domain
FP	Fabry–Pérot
FWHM	Full width at half maximum
HF	Hydrofluoric acid
IUPAC	International Union of Pure and Applied Chemistry
β-LG	β-Lactoglobulin
PDMS	Polydimethylsiloxane
PMMA	Polymethyl methacrylate
PMLs	Perfectly matched layers
pSi	Porous silicon
pSiMC	Porous silicon microcavity
PTFE	Polytetrafluoroethylene
R6G	Rhodamine 6G
SEM	Scanning electron microscopy
TMM	The transfer matrix model
QF	Quality factor
QD	Quantum dot
WGM	Whispering-gallery mode
FTIR	Fourier transform infrared
NIR	Near-Infrared
