# Supplementary material for: Enhanced Light–Matter Interaction in Porous Silicon Microcavities Structurally Optimized Using Theoretical Simulation and Experimental Validation
Source: Nanomaterials (Basel). 2025 Nov 29;15(23):1808. doi: 10.3390/nano15231808 (PMC12693180; doi:10.3390/nano15231808)
Supplement: Supplementary file 1 [file nanomaterials-15-01808-s001.zip › nanomaterials-3989915-SM-final.pdf]

SUPPORTING INFORMATION

Enhanced Light–Matter Interaction in Porous Silicon Microcavities Structurally Optimized Using Theoretical Simulation and Experimental Validation

Evelyn Granizo [1], Irina S. Kriukova [1,2], Aleksandr A. Knysh [1,2], Pavel M. Sokolov [1,2,3], Pavel S. Samokhvalov [1,2] and Igor R. Nabiev [1,3,4,*]

[1] Research Center Nano-Photon, National Research Nuclear University MEPhI (Moscow Engineering Physics Institute), 115409 Moscow, Russia; aleroman16@hotmail.com (E.G.)
[2] Life Improvement by Future Technologies (LIFT) Center, 121205 Moscow, Russia; i.krukova@lift.center (I.K.);
[3] Department of Clinical Immunology and Allergology, Sechenov First Moscow State Medical University (Sechenov University), 119146 Moscow, Russia;
[4] Université de Reims Champagne-Ardenne, BioSpecT UR-7504, 51100 Reims, France; igor.nabiev@univ-reims.fr (I.N.)
* Correspondence: igor.nabiev@univ-reims.fr

SECTION S1.
Approximation Functions for Refractive Indices of Si and SiO_2

Theoretical models are essential for designing pSiMCs with predictable optical properties because they can be used to determine the optimal parameters of the cavity structure. In general, the use of a theoretical model for selecting the parameters of pSi etching can be described as follows. Once the desired cavity resonance wavelength λ is selected, theoretical models are used to determine the porosities and refractive indices of the layers that are required for obtaining the refractive index contrast that ensures confinement of light with the given λ. Then, the minimum and maximum currents for the electrochemical etching are selected to obtain the layers with these porosities and refractive indices. Specifically, a low-porosity layer with a high refractive index is formed if a low etching current is applied, whereas a high current results in a high-porosity layer with a low refractive index. The thicknesses of the layers are calculated from their selected effective refractive indices so that the resonance condition is met: $d = \lambda/4n$, where d is the layer thickness, λ is the wavelength of light, and n is the refractive index. The time intervals required for etching porous layers with the calculated thicknesses are estimated from calibration curves obtained in a series of preliminary experiments. Additionally, the theoretical reflection spectrum of the pSiMC provides data on some key optical parameters, such as the full width at half maximum (FWHM) of the cavity mode, which helps estimating the QF. Thus, the results of theoretical simulation guide the design of pSiMCs, optimize the etching procedure, and aid in better understanding the microcavity structure and predicting pSiMC performance before fabrication. The accuracy of these results depends on the input data, such as the complex refractive index of silicon.

For precise calculations using theoretical models and for calibration of the etching parameters and further optimization of the pSiMC structure, an approximation of the dependence of the complex refractive index on the wavelength is used. The real component of the complex refractive index describes the refraction of light. It is equal to the ratio of the speed of light in vacuum to the speed of light in silicon. The imaginary component (κ) describes the absorption or attenuation of light. For silicon, the imaginary component is particularly important in the visible and infrared ranges, because silicon strongly absorbs

light at shorter wavelengths but is more transparent at longer wavelengths [110]. The dependence of the refractive index of silicon oxide on the wavelength is well approximated by the Sellmeier equations [111]. In the visible and near-infrared spectral regions, silicon oxide has negligible absorption.

The absorption spectrum of silicon in the theoretical model of a microcavity is determined by the complex refractive index, $RI = n + i\kappa$. The real component of the refractive index, n, describes the refraction of light and is equal to the ratio of the speed of light in vacuum to the speed of light in silicon. The imaginary component, κ, is the absorption coefficient of silicon (Figure S1).

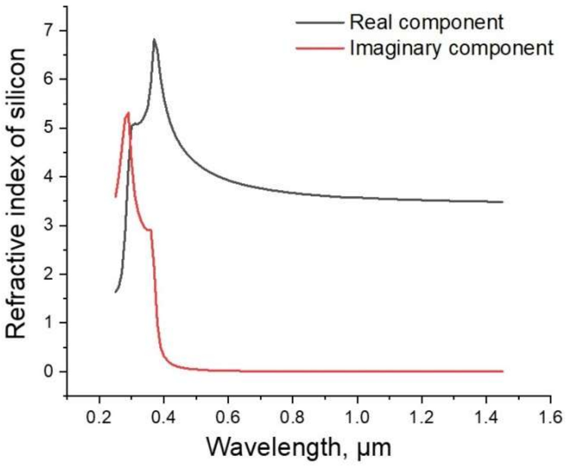

Figure S1. The real (black curve) and imaginary (red curve) components of the refractive index of silicon as a function of the wavelength.

The dependences of the refractive index on the porosity and oxidation at different wavelengths have been calculated using the effective medium approximation. The relationship between the real component of the silicon refractive index (n_{Sical}) and the wavelength can be well approximated by the Sellmeier equations:

$$n_{\mathrm{Sical}}(\lambda) = 36036 \cdot e^{\left(\frac{-\lambda}{37.8}\right)} + 11.96 \cdot e^{\left(\frac{-\lambda}{152}\right)} + 0.892 \cdot e^{\left(\frac{-\lambda}{499}\right)} + 3.435. \quad (1)$$

The imaginary component of the silicon refractive index is a complex function (κ_{Sical}) that has been defined in the range from 380 to 1000 nm:

$$\kappa_{\mathrm{Sical}}(\lambda) = \left| \begin{array}{c} 1.7365810^{13} \cdot e^{\left(\frac{-\lambda}{12.3403}\right)} + 0.14788 \quad for\ \lambda \in [380; 420); \\ 6855.42451 \cdot e^{\left(\frac{-\lambda}{38.90007}\right)} + 0.02607 \quad for\ \lambda \in [420; 520); \\ 0.96502 \cdot e^{\left(\frac{-\lambda}{156.96774}\right)} - 7.3990510^{-4} \quad for\ \lambda \in [520; 1000); \\ 0 \quad otherwise. \end{array} \right. \quad (2)$$

Therefore, the total function of the complex refractive index of silicon (n_{TSical}) is

$$n_{\mathrm{TSical}}(\lambda) = n_{\mathrm{Sical}}(\lambda) + i \cdot \kappa_{\mathrm{Sical}}(\lambda). \quad (3)$$

The approximation function for silicon dioxide ($n_{\mathrm{SiO_2cal}}$) is [1]

$$n_{\mathrm{SiO_2cal}}(\lambda) = \left[\left[\frac{0.6961663 \cdot \lambda^2}{\lambda^2 - (68.4043)^2} + \frac{0.4079426 \cdot \lambda^2}{\lambda^2 - (116.2414)^2}\right] + \frac{0.8974794 \cdot \lambda^2}{\lambda^2 - (9896.161)^2} + 1\right]^{1/2} \quad (4)$$

$$\kappa_{\mathrm{SiO_2cal}}(\lambda) = 0. \quad (5)$$

It is essential that a theoretical model of the optical properties of pSiMCs correctly takes into account the material porosity. Because pSi is a combination of crystalline silicon, air-filled pores, and a thin layer of silicon oxide at the interface, effective medium approximations are often used to estimate the refractive index of the complex structure. In the case of pSi, Bruggeman effective medium approximation is commonly assumed to correctly predict the effective refractive index on the basis of the porosity and the material composition, namely, the proportions of Si, SiO_2, and air or solvent trapped in the pores.

SECTION S2.
Bruggeman Effective Medium Approximation

The effective refractive index describes the optical behavior of a composite material as if it were a homogeneous medium. Here, we use the Bruggeman effective medium approximation to estimate the effective refractive index of pSi layers that form the microcavity. In the case of pSi, the effective refractive index depends on the volume fractions of its three constituents: silicon (Si), air-filled pores, and silicon dioxide (SiO_2) formed on the pore surface due to spontaneous oxidation of silicon [80]. Therefore, in our case, the Bruggeman effective medium approximation is given by the equation:

$$f_{Air} * \left(\frac{\varepsilon_{Air} - \varepsilon_{eff}}{\varepsilon_{Air} + 2\varepsilon_{eff}}\right) + f_{Si} * \left(\frac{\varepsilon_{Si} - \varepsilon_{eff}}{\varepsilon_{Si} + 2\varepsilon_{eff}}\right) + f_{SiO_2} * \left(\frac{\varepsilon_{SiO_2} - \varepsilon_{eff}}{\varepsilon_{SiO_2} + 2\varepsilon_{eff}}\right) = 0, \quad (6)$$

where f_{Air}, f_{Si}, and f_{SiO_2} are the volume fractions of air, Si, and SiO_2; ε_{Air}, ε_{Si}, and ε_{SiO_2} are their dielectric constants ($\varepsilon = n^2$), and ε_{eff} is the effective dielectric constant of the pSi layer. The sum of volume fractions satisfies the condition:

$$f_{Air} + f_{Si} + f_{SiO_2} = 1. \quad (7)$$

Equation (6) can be solved in two steps. First, the effective refractive index of the silicon/silicon oxide medium is calculated. Then, this result is used to determine the final effective refractive index for the air-filled porous material.

Section 2.1.
Effective Dielectric Constant of the Si/SiO2 Medium

Porous silicon undergoes oxidation over time; therefore, we first consider a model of the two-component Si/SiO_2 material and then move to the porous material. At this step, we approximate the effective dielectric constant (ε_1) of the Si/SiO_2 mixture using the Bruggeman equation:

$$f_{Si} * \left(\frac{\varepsilon_{Si} - \varepsilon_1}{\varepsilon_{Si} + 2\varepsilon_1}\right) + f_{SiO_2} * \left(\frac{\varepsilon_{SiO_2} - \varepsilon_1}{\varepsilon_{SiO_2} + 2\varepsilon_1}\right) = 0, \quad (8)$$

where ε_1 is the effective dielectric constant of the Si/SiO_2 medium, and $f_{Si} + f_{SiO_2} = 1$.

Section 2.2.
Final Effective Refractive Index for the Porous Material

Once the effective dielectric constant ε_1 of the Si/SiO_2 medium is determined, the effect of air-filled pores is added into the second Bruggeman equation:

$$f_{Air} * \left(\frac{\varepsilon_{Air} - \varepsilon_{eff}}{\varepsilon_{Air} + 2\varepsilon_{eff}}\right) + f_1 * \left(\frac{\varepsilon_1 - \varepsilon_{eff}}{\varepsilon_1 + 2\varepsilon_{eff}}\right) = 0, \quad (9)$$

where $f_1 = f_{Air} + f_{SiO_2}$ (the combined fraction of silicon and silicon oxide) and ε_{Air} is the dielectric constant of air (≈ 1). Once ε_{eff} is determined, the effective refractive index (n_{eff}) is calculated as $n_{eff} = \sqrt{\varepsilon_{eff}}$.

The effective refractive index for the given porosity having been estimated, the layer thickness that would ensure the desired spectral position of the microcavity mode is determined from the quarter-wavelength condition. Then, the etching rate calibration curves obtained in preliminary experiments are used to calculate the etching times as $t = d/v$, where d is the layer thickness and v is the etching rate. The methods used to determine the etching rates from experimental data and construct the calibration curves.

Thus, the Bruggeman effective medium approximation provides a reliable method for estimating the refractive index of pSi layers from their porosity and oxidation state. However, more detailed predictions of the optical characteristics of multilayer pSi structures require additional theoretical models. One of these is the transfer matrix model, which allows the simulation of light propagation through layered media and prediction of its optical response.

SECTION S3.
Transfer Matrix Model

The transfer matrix model (TMM) allows theoretical simulation of the optical properties of single-layer and multilayer structures, including DBRs and microcavities [112]. This method is applicable only to 1D layered structures, where the refractive index varies in a single direction. To illustrate the method, let us consider a plane wave incident perpendicularly onto the surface of a multilayer structure (Figure S2). The light wave undergoes partial reflection and transmission at every interface, generating an infinite sequence of multiple reflections and transmissions within the structure. It is assumed that only two counter-propagating waves exist in each layer, one traveling forward and the other backward. By summation of the contributions of the waves traveling in the same direction, the system can be described using two complex amplitude components, U^+ and U^-, which represent plane waves propagating in opposite directions perpendicular to the multilayer plane. Consequently, description of the light propagation through a multilayer medium is reduced to determination of the amplitudes of these two waves propagating through each layer of the pSiMC.

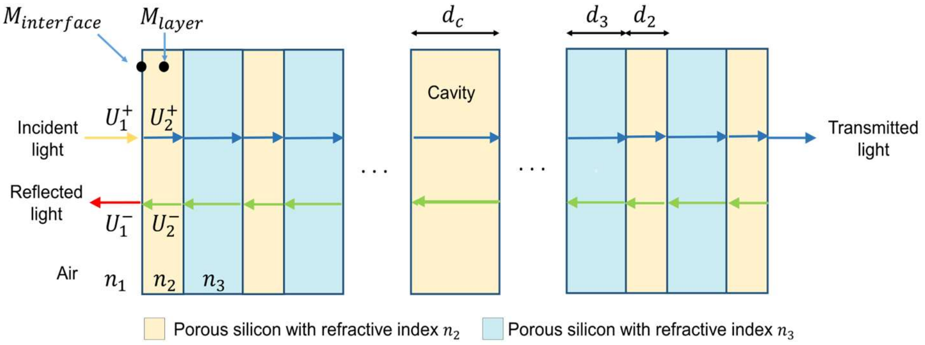

Figure S2. Schematic representation of light propagation through a porous silicon microcavity.

Then, an electromagnetic wave passing through the interface of two layers with different refractive indices (e.g., air and pSi layer with refractive index n2, see Figure S2) can be described as

$$\begin{bmatrix} U_2^{(+)} \\ U_2^{(-)} \end{bmatrix} = M \begin{bmatrix} U_1^{(+)} \\ U_1^{(-)} \end{bmatrix}, \tag{10}$$

where U_1^+ and U_1^- are the complex amplitudes of the wave in the layer before the interface (the layer with the refractive index n_1), U_2^+ and U_2^- are the complex amplitudes of the wave

in the second layer (with the refractive index n_2), and M is the transfer matrix that relates the amplitudes before and after the interface.

For a system composed of multiple layers with different refractive indices and thicknesses, two types of matrices are used: a homogeneous layer matrix and an interface matrix. The homogeneous layer matrix ($M_{L,j}$) describes how the wave propagates within a layer with the thickness d_j and the refractive index n_j:

$$M_{\mathrm{L,j}} = \begin{bmatrix} e^{ik_j d_j} & 0 \\ 0 & e^{-ik_j d_j} \end{bmatrix}, \tag{11}$$

where $k_j = \frac{2\pi n_j}{\lambda}$ is the wavevector in the layer.

The interface matrix ($M_{I,j}$) describes how the wave is transmitted and reflected at the boundary between two adjacent layers:

$$M_{\mathrm{I,j}} = \frac{1}{2}\begin{bmatrix} 1 + \frac{n_{j+1}}{n_j} & 1 - \frac{n_{j+1}}{n_j} \\ 1 - \frac{n_{j+1}}{n_j} & 1 + \frac{n_{j+1}}{n_j} \end{bmatrix}, \tag{12}$$

where n_j is the refractive index of the layer j and n_{j+1} is the refractive index of the layer j+1.

A multilayer medium, such as pSiMC, consisting of N elements is characterized by the product of the transfer matrices of each individual element. Then, the total transfer matrix (M_{total}) is obtained by multiplying the matrices for all layers and interfaces:

$$M_{\mathrm{total}} = \prod_{j=1}^{N} (M_{I,j} M_{L,j}), \tag{13}$$

where, $M_{I,j}$ is the interface matrix between layers j and j+1; $M_{L,j}$ is the layer matrix in the layer j; N is the total number of layers. The total transfer matrix

$$M_{\mathrm{total}} = \begin{pmatrix} m_{11} & m_{12} \\ m_{21} & m_{22} \end{pmatrix}, \tag{14}$$

is used to calculate the reflection (R) and transmission (T) coefficients:

$$T = \left| \frac{m_{11}m_{22} - m_{12}m_{21}}{m_{22}} \right|^2, R = \left| \frac{m_{21}}{m_{22}} \right|^2. \tag{15}$$

Computational implementation of the described approach using various mathematical software packages [113–115] allows accurate calculation of the reflection and transmission spectra of multilayer structures [81,82], such as pSiMCs and DBRs, and enables the design of optical components with desired optical properties.

Figure S3 demonstrates excellent agreement between the reflectance spectrum of a pSiMC calculated using the TMM and that measured experimentally, particularly in predicting the spectral position of the MC eigenmode and the general shape of the spectrum. Minor differences in the FWHM of the eigenmode and the photonic band gap can be explained by that the interfaces between the layers forming the real pSiMC were not ideally smooth. Thus, we consider the TMM to be the fastest, most straightforward, and most reliable simulation method. It was our primary tool for estimating the pSiMC structural parameters and expected optical characteristics.

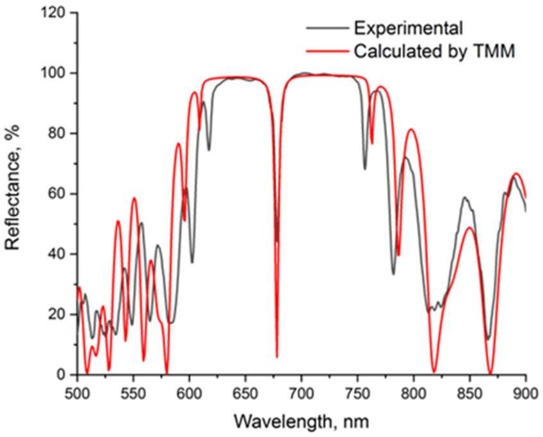

Figure S3. Comparison of reflectance spectra of a porous silicon microcavity obtained experimentally (grey curve) and calculated using the transfer matrix model (red curve).

In summary, our basic theoretical approach combines the Bruggeman effective medium model with the TMM. The complex refractive indices and SEM analysis of the structural parameters of the pSi layers that was performed in preliminary experiments on etching rate calibration serve as input parameters for calculating the quarter-wave optical path of pSi layers so as to maximize the reflectivity in the specified range and to obtain the desired cavity eigenmode. This approach enables precise determination of layer etching times and the resulting optical spectra through computation of the M_{total} matrix.

Although TMM is a fast and efficient analytical method for calculating the parameters of waves propagating through the multilayer structure of Bragg reflectors and 1D MCs, it has inherent limitations. This model assumes idealized multilayer photonic structures that have perfectly aligned, flat interfaces and a single direction of wave propagation. In addition, TMM may give inaccurate results when applied to materials with strong absorption or high scattering. Numerical methods, such as the finite element method (FEM) and the finite-difference time-domain (FDTD) method, are free from these limitations. Their applicability to pSiMCs is discussed below.

SECTION S4.
Numerical Methods

Numerical methods, such as the FDTD and FEM are techniques for solving Maxwell's equations that are used in analysis of electromagnetic wave propagation in complex two-dimensional (2D) and three-dimensional (3D) multimaterials [83,84]. These methods accurately simulate processes in strongly scattering, resonant, or highly absorptive materials, which makes them essential for studying pSiMCs. Both methods are based on the subdivision of the entire structure to be simulated and analyzed (the computational domain) into smaller elements to obtain approximate solution, differing in the type of these elements. Simulation software have been developed for both methods, the only inconvenience being that the data on the pSi refractive indices should be introduced into the calculation manually, because their values depend on the porosity and, hence, no database on pSi refractive indices is available (in contrast to most common materials).

Calculation by the FDTD method employs a structured Cartesian grid consisting of rectangular (2D) or cubic (3D) cells, which can hinder accurate simulation of curved surfaces. FDTD also implies time discretization, which is useful for simulation of wave propagation and study of time-dependent phenomena. Because this is a time-domain method, a single simulation can capture responses across a wide range of frequencies, making it

efficient for broadband analysis. Additionally, FDTD can include nonlinearity in material properties during the discretization process, thereby enabling simulation of complex interactions. On the other hand, FDTD simulation of the electromagnetic wave propagation yields only approximated rather than exact solutions of Maxwell's equations, because a discrete grid used for calculations. This leads to numerical dispersion error, which accumulates with time and can affect the accuracy of long-term simulations. This numerical dispersion is a major limitation of the FDTD method.

To illustrate the use of the FDTD method for simulating complex light–matter interaction, we simulated the luminescence of luminophores embedded in the cavity layer of a pSiMC (Figure S4a). The microcavity under study was made of a top DBR consisting of six pairs of quarter-wave layers with low (L) and high (H) porosities, a double (half-wave) L cavity layer, and a bottom DBR consisting of ten pairs of H and L layers: $(LH)_6L_2(HL)_{10}$. Based on the results of transfer matrix calculations, the thicknesses of the pSiMC layers were selected so that the spectral position of the cavity eigenmode coincided with the luminescence maximum of the luminophore (FWHM = 40 nm, centered at 568 nm) in order to maximize the interaction between light and matter. FDTD simulations showed a 14.3-fold narrowing of the emission spectrum in the hybrid structure down to 2.8 nm, i.e., to about the width of the pSiMC eigenmode (Figure S4b). The same effect has been observed experimentally for various emitters placed inside a pSiMC [43,116].

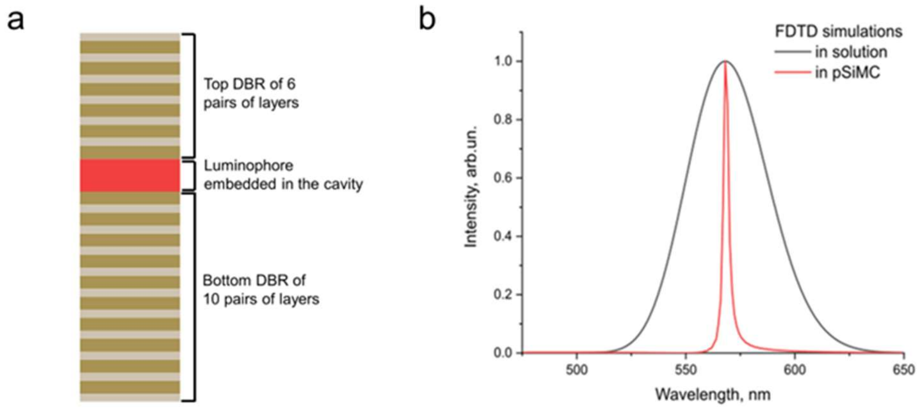

Figure S4. (a) Schematic representation of a porous silicon microcavity simulated using the finite-difference time-domain (FDTD) method. The microcavity consists of a top distributed Bragg reflector (DBR) formed by six pairs of low (L) and high (H) porosity quarter-wave layers, a cavity representing a double (half-wave) L layer, and a bottom DBR formed by ten pairs of H and L layers: $(LH)_6L_2(HL)_{10}$. The luminophore is embedded in the central cavity. (b) Luminescence spectra of the luminophore in a solution (black curve) and in the microcavity (red curve).

The FEM entails spatial discretization of the system into finite elements, and it can only be used to simulate static states, but not processes evolving with time. However, in contrast to the FDTD method, the FEM is applicable to complex geometries and inhomogeneous materials, because the shape of the finite elements can be irregular, based on triangular or tetrahedral discretization. Generally, the approximation quality in the FEM is higher than in the FDTD method, but it strongly depends on the problem to be solved; therefore, this does not overwhelm the advantages of the FDTD method. However, FEM is well suited for solving interdisciplinary problems combining, e.g., electromagnetism with heat transfer, hydrodynamics, and elasticity. Figure S5 shows an example of FEM simulation of an $(LH)_NL_2(HL)_N$ pSiMC.

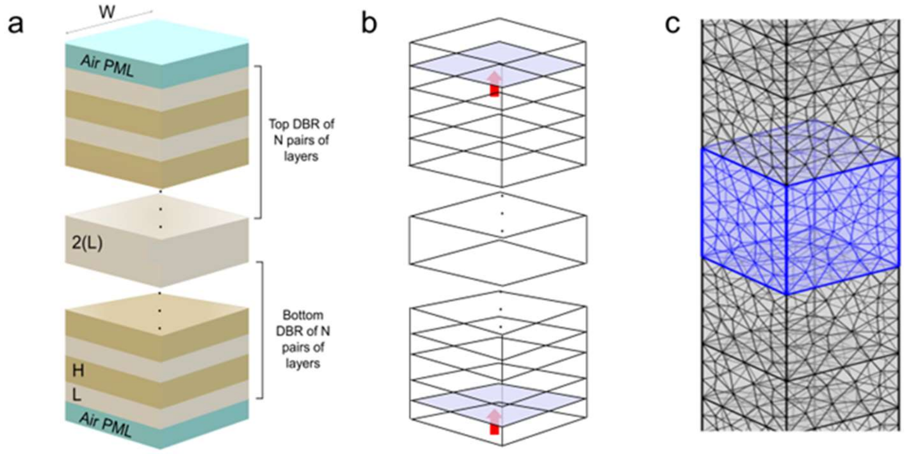

Figure S5. Schematic representation of a porous silicon microcavity (pSiMC) simulated using the finite element method. (a) The microcavity consists of two distributed Bragg reflectors (DBRs), a central cavity, and two perfectly matched layers (PMLs). The DBRs are composed of *N* pairs of alternating layers with low (L) and high (H) porosities, refractive indices n_L and n_H, and thicknesses d_L and d_H, respectively. The cavity, located between the DBRs, has a refractive index of n_L and a thickness of $2d_L$. *W* is the lateral dimension of the pSiMC. (b) Simulation ports with the direction of light propagation indicated. (c) A section of the mesh generated inside the microcavity.

The computational tasks in this FEM simulation include the selection of the optical properties of the materials, determining the geometry of the structure, setting the boundary conditions, and subdivision of the structure into smaller regions for numerical analysis. The pSiMC shown in Figure S5 consists of two DBRs made of alternating low- and high-porosity layers with high and low refractive indices, respectively, and a cavity layer between them. The effective refractive indices of low- and high-porosity layers are calculated using the Bruggeman effective medium approximation. By introducing the effective refractive indices and the eigenmode λ, the thicknesses of the DBRs layers are selected to match the quarter-wavelength ($\lambda/4n$) condition, the thickness of the cavity layer being $\lambda/2n_L$. These conditions ensure that the DBRs reflect light efficiently. Alternatively, the layer thicknesses of an experimental pSiMC sample, estimated from SEM analysis, can be introduced into the simulation.

For a more correct simulation of the light interaction with the structure, boundary layers called perfectly matched layers (PMLs) are added at the top and bottom of the pSiMC (Figure S5a). These PMLs truncate the unbounded computational region by absorbing the outgoing waves and prevent artificial reflections from the edges of the computational domain that would otherwise interfere with the results. Additionally, scattering boundary conditions are applied at the outer edge of the system to allow any remaining waves to exit the system without reflection. These conditions are essential for simulation of an open optical system and accurately represent how light would behave in a real environment. In selecting the materials for the components of the microcavity structure, it is important to manually set the refractive indices of the constituting porous layers, because they differ from the standard values for raw materials typically used by default in simulation software. In addition, the imaginary part of the refractive index, which represents energy loss, should also be taken into account, because, in a real microcavity, light is partly absorbed by the material.

Note that dividing the simulated structure into small elements, known as mesh, allows the numerical method to approximate the solution of Maxwell's equations throughout the structure (Figure S5c). The size of the small elements affects the balance between

the accuracy of simulation results and the computation time, making the selection of the optimal mesh size essential.

In order to simplify the numerical simulation of periodic multilayer structures and reduce the calculation time, the periodic boundary conditions (e.g., Floquet conditions) are applied in the lateral direction in order to represent the structure as a repetitive unit. The interaction between pSiMC and light is simulated by introducing input and output regions (ports) at the bottom and top of the structure, respectively (Figure S5b). The light wave enters the structure from the bottom along the z direction with a specified electric field amplitude (e.g., $E(x) = 1\ V/m$) and exits from the top. The evaluation of the system is performed only in the wavelength range of interest, to avoid unreasonable extension of the computation time. The simulation results allow visualization of the confinement of the electric field within the cavity. Calculations for more complex systems, e.g., structures with embedded nanoparticles, can be performed in 3D simulations [117].

We used our pSiMC structure model shown in Figure S5 to perform FEM simulations in order to determine the effect of the porosity (L or H) of the cavity layer on the field distribution within the microcavity. Figures S6a and S6b show the field distribution in $(LH)_6L_2(HL)_{10}$ and $(HL)_6H_2(LH)_{10}$ microcavities; i.e., the cavity was a double-thickness ($d = \lambda/2n$) layer with a low or high porosity, respectively. The simulations were performed for a pSiMC with an eigenmode at $\lambda = 560$ nm. Simulations have shown that the field is better confined and concentrated about the center of the high-porosity cavity. In the case of a low-porosity cavity, the field is concentrated at the boundaries of the cavity. The main difference in field distribution between these two configurations that is observed experimentally is a deeper reflectance dip at the position of the eigenmode of the pSiMC with the high-porosity cavity layer (Figure S6c).

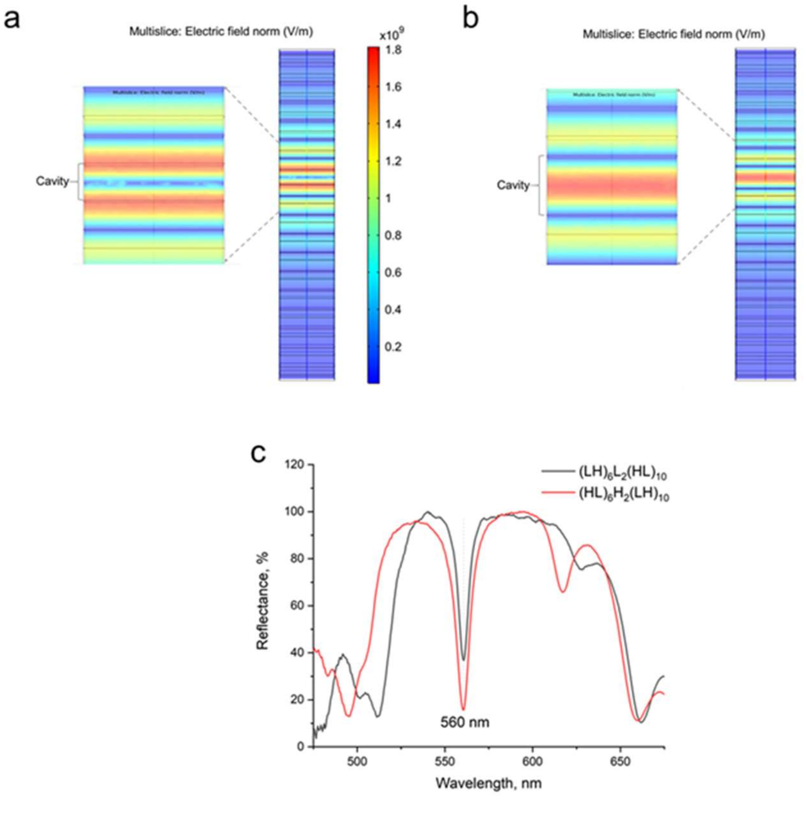

Figure S6. FEM simulations of the electric field distribution in the (a) $(LH)_6L_2(HL)_{10}$ and (b) $(HL)_6H_2(LH)_{10}$ porous silicon microcavities, where L and H are low- and high-porosity layers, respectively. (c) Reflectance spectra of experimentally fabricated $(LH)_6L_2(HL)_{10}$ (black) and $(HL)_6H_2(LH)_{10}$ (red) porous silicon microcavities.

In summary, the analytical transfer matrix method and numerical methods allow better understanding of the relationships between pSiMC structure and its optical properties. Both approaches are essential in studies of wave propagation. The TMM remains the preferred option, offering rapid spectral analysis of ideal 1D layered systems if the effective medium approximation is also used for estimating the effective refractive indices of pSi layers. However, inherent limitations of the TMM, including its restriction to planar geometries, necessitate alternative approaches for more sophisticated systems. FEM and FDTD simulations are used to overcome these limitations by solving Maxwell's equations in the time and frequency domains. The FDTD method excels in understanding light–matter interactions extended in time, which allows the study of near and far fields, and the FEM enables the study of various photonic systems with intricate geometries. However, simulations using Maxwell's equations are computationally intensive, often requiring substantial memory and processing power, especially when dealing with 3D structures. However, strategic use of unit cells in simulation and careful selection of the task-appropriate ranges of system parameters can boost the efficiency of simulations. The choice between analytical and numerical methods ultimately depends on the specific physical phenomena under investigation and the available computational resources.

SECTION S5.
SEM Images of Porous Silicon Monolayers and a Photograph of Porous Silicon Microcavities

The surface and cross-sectional SEM images of porous silicon monolayers are shown in Figures S7 and S8, respectively. The porous silicon samples were fabricated by electrochemical etching on a single-sided polished p^+-type silicon wafer (100) with a resistivity of 0.001–0.005 Ω·cm. A photograph of porous silicon microcavities is presented in Figure S9. The color is a characteristic feature that indicates the spectral region of the resonant mode of the microcavity.

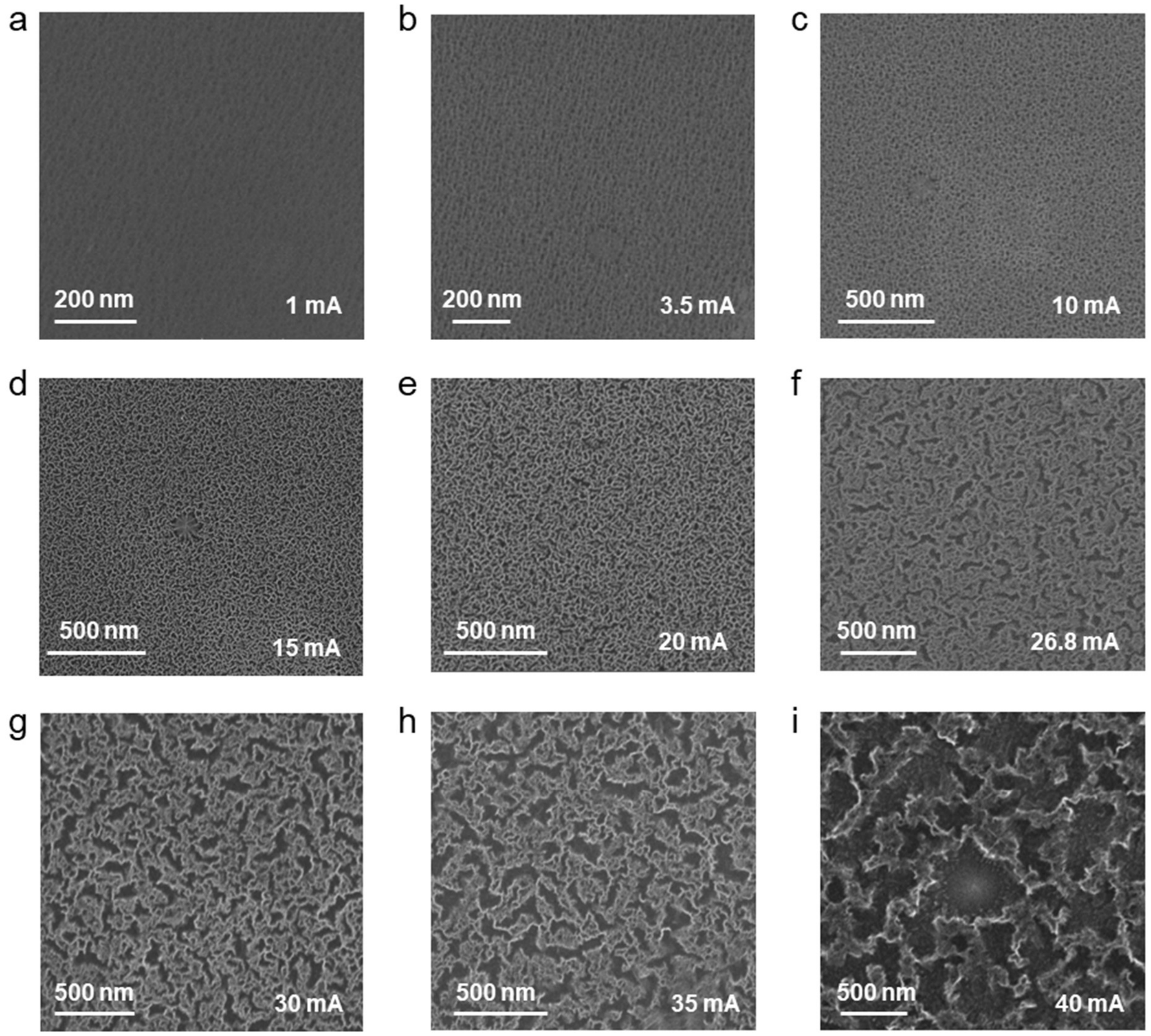

Figure S7. Surface SEM images of porous silicon monolayers with different porosities, corresponding to etching currents ranging from 1 to 40 mA.

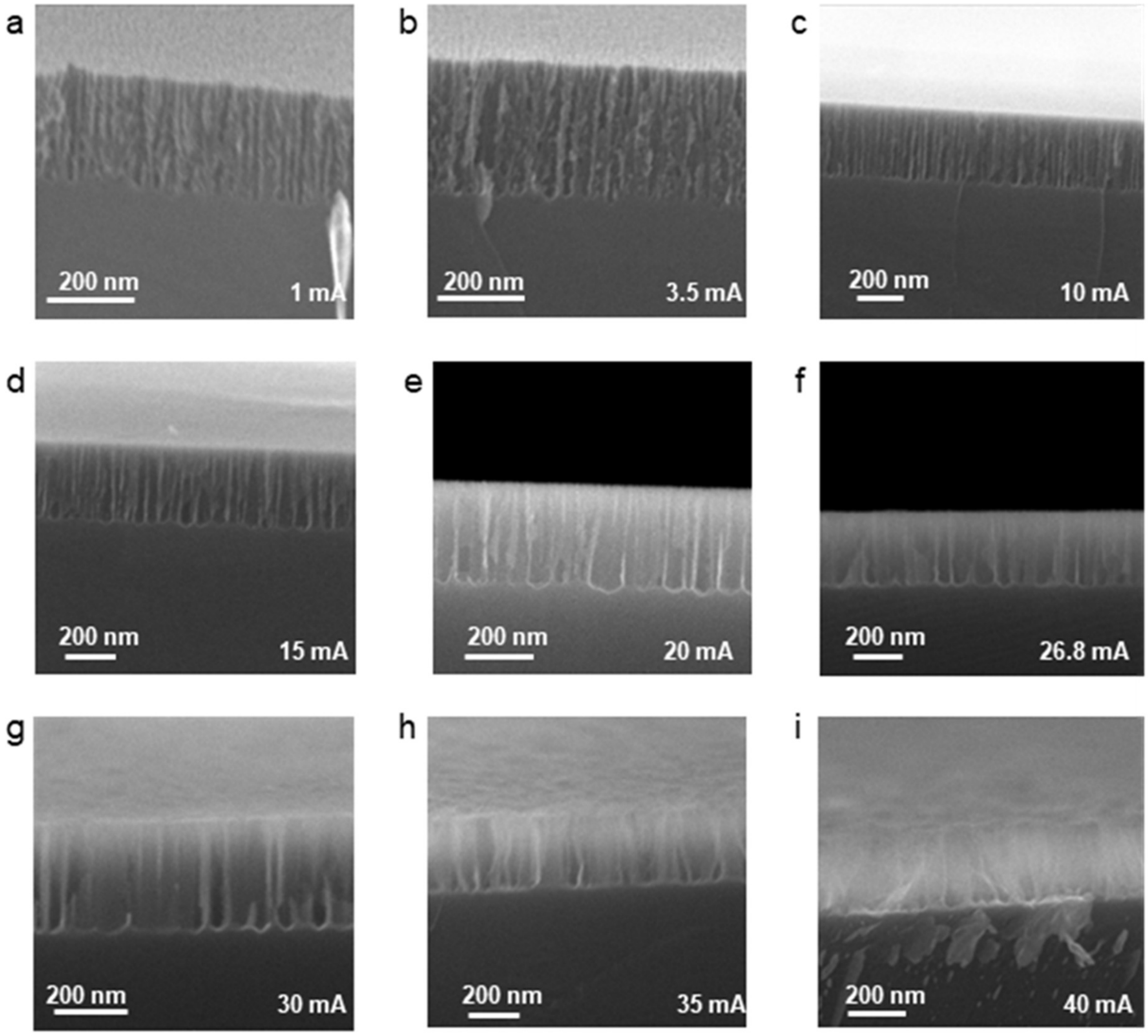

Figure S8. Cross-sectional SEM images of porous silicon monolayers with different porosities, corresponding to etching currents ranging from 1 to 40 mA.

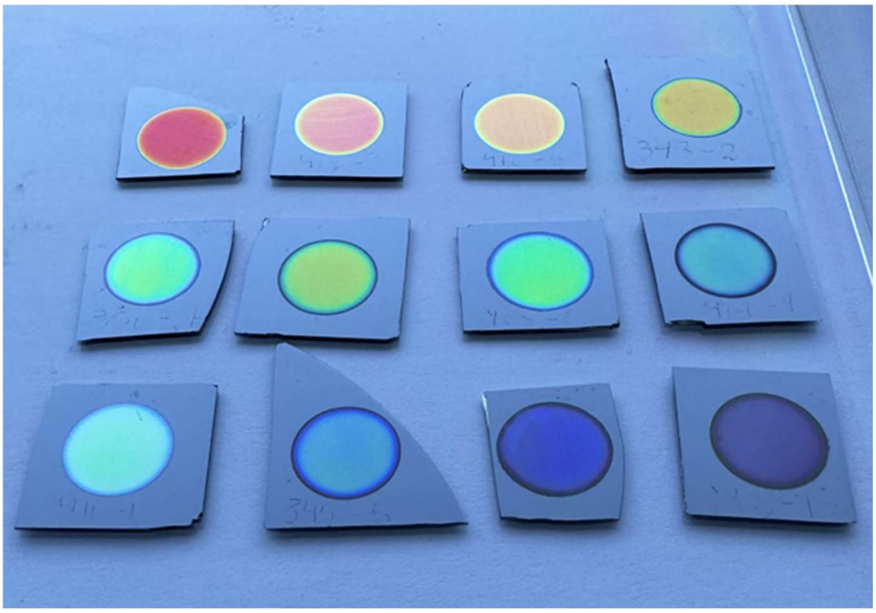

Figure S9. Photograph of the porous silicon microcavities.

SECTION S6.
Troubleshooting and Safety Considerations

6.1. Troubleshooting

Common troubleshooting strategies and essential safety precautions should be considered to ensure reproducible properties and safe operation during the electrochemical etching of porous silicon.

1. Substrate breakage during cutting. The substrate breaks into too small pieces during cutting of a Si wafer.
Cause: Poor cutting technique.
Solutions: Check the crystallographic orientation of the wafers and the cutting technique. In the case of (100) wafers, place the entire wafer on a stable surface, e.g., a thick PTFE plate, with the flat edge facing you. Cut half of the substrate by placing the cutter on the flat edge and applying a slight pressure. Repeat the procedure for the two halves to obtain the substrates of the required size.

2. Nonuniform pore formation. Irregular pore distribution over the substrate surface.
Causes: Poor electrical contact with the Si wafer; nonuniform HF concentration; unstable etching current.
Solutions: Ensure that the cables are connected correctly to the power source and are in good contact with the platinum cathode and the aluminum anode; ensure a uniform concentration of HF by mixing the electrolyte thoroughly; ensure the correct and stable power supply.

3. Unstable etching. Variation of the pore size and layer thickness across the microcavity in the lateral direction.
Causes: Depletion of the etching solution in HF; accumulation of hydrogen bubbles on the surface.
Solutions: Fine-tune the etching current, use the gradient function for the etching time to correct the variation of layer thickness, and use pauses after etching of each layer for the dissipation of the evolving H_2 bubbles.

4. Eye-visible defects on the etched Si surface. Small dots on the surface indicate substrate or reagent contamination.
Causes: Contamination of the substrates due to poor cleaning, accumulation of dust or small Si fragments on the substrate surface; contaminated reagents; improper storage of the etching cell allowing exposure to dust.
Solutions: Clean and dry substrates using pure solvents, in a clean, dust-free environment; store the etching cell and substrate containers properly to avoid contamination. Try an alternative procedure for chemical cleaning of the substrate, e.g., using a KOH solution.

5. Cracking or detachment of the porous layer. Porous layer peels off or cracks during sample processing or after mechanical damage.
Cause 1: An excessive etching depth; excessive capillary stresses caused by water evaporation; high etching current densities.
Solutions: Reduce the etching times; use a correct technique to rinse and dry the substrate (e.g., using a liquid with a lower surface tension than that of water; use supercritical or freeze-drying). Check that the etching current does not reach the critical values which would switch the process to the electropolishing regime.
Cause 2: Physical contact of the pipette tip with the porous surface during removal of the etching solution; improper handling of samples using instruments (tweezers, etc.); scratching of surface with instruments; dropping of the substrate on contaminated surfaces; over-tightening of the screws clamping the cell.
Solutions: Handle the pipettes and tweezers carefully; secure the substrate gently inside the cell; avoid unnecessary mechanical stresses on the substrate and etching cell during its assembly and handling.

5. Drift of the reflectance spectrum over the sample surface. Drift of the reflectance spectrum at some sites on the sample with a slight change in the eigenmode properties.
Cause: Irregular interfaces between the layers of the microcavity and irregular layer porosity.
Solution: Carefully select substrates with the required resistivity and crystallographic orientation; perform thorough calibration of the layer porosity and etching rate and adjust the etching parameters according to the results.

6.2. Safety Considerations

1. Hydrofluoric acid (HF). Handle in a certified well-ventilated fume hood; wear a full set of personal protective equipment: HF-resistant (e.g., neoprene or nitrile) gloves, a lab coat, goggles or a face shield. Use containers made of HF-resistant materials (e.g., polytetrafluoroethylene or polypropylene). Avoid using glassware.
2. Hydrogen gas evolution. Ensure adequate ventilation of the etching setup area; avoid open flames and sparks due to the risk of inflammation or explosion of the hydrogen gas generated during etching.
3. Electrical hazards. Use shielded connectors and insulated wiring; avoid contacts of the electrolyte with the equipment.
4. Waste disposal. HF-containing waste should be collected in labeled, suitable containers and disposed properly.
5. Sample handling. Handle the pSi samples gently, because they are mechanically fragile and can generate fine dust when dry; avoid grinding or fracturing them without protective measures.

References

43. Kriukova, I.S.; Granizo, E.A.; Knysh, A.A.; Samokhvalov, P.S.; Nabiev, I.R. Controlling the Luminescence of Quantum Dots in Hybrid Structures Based on Porous Silicon. *Phys. At. Nucl.* **2024**, *87*, 1750–1753. https://doi.org/10.1134/S1063778824100259.
80. Bruggeman, D.A.G. Berechnung Verschiedener Physikalischer Konstanten von Heterogenen Substanzen. I. Dielektrizitätskonstanten Und Leitfähigkeiten Der Mischkörper Aus Isotropen Substanzen. *Ann. Der Phys.* **1935**, *416*, 636–664. https://doi.org/10.1002/andp.19354160705.
81. Swe, S.K.; Noh, H. Inverse Design of Reflectionless Thin-Film Multilayers with Optical Absorption Utilizing Tandem Neural Network. *Photonics* **2024**, *11*, 964. https://doi.org/10.3390/photonics11100964.
82. Fedorova, I.V.; Eliseeva, S.V.; Sementsov, D.I. Transmission and Reflection Spectra of a Bragg Microcavity Filled with a Periodic Graphene-Containing Structure. *Appl. Sci.* **2023**, *13*, 7559. https://doi.org/10.3390/app13137559.
83. Pepper, D.W.; Heinrich, J.C. The Finite Element Method: Basic Concepts and Applications with MATLAB®, MAPLE, and COMSOL, 3rd ed.; CRC Press: Boca Raton, FL, USA, 2017; ISBN 978-1-315-39510-4.
84. Oskooi, A.F.; Roundy, D.; Ibanescu, M.; Bermel, P.; Joannopoulos, J.D.; Johnson, S.G. Meep: A Flexible Free-Software Package for Electromagnetic Simulations by the FDTD Method. *Comput. Phys. Commun.* **2010**, *181*, 687–702. https://doi.org/10.1016/j.cpc.2009.11.008.
110. Aspnes, D.E.; Studna, A.A. Dielectric Functions and Optical Parameters of Si, Ge, GaP, GaAs, GaSb, InP, InAs, and InSb from 1.5 to 6.0 eV. *Phys. Rev. B* **1983**, 27, 985–1009. https://doi.org/10.1103/PhysRevB.27.985.
111. Malitson, I.H. Interspecimen Comparison of the Refractive Index of Fused Silica*,†. *J. Opt. Soc. Am.* **1965**, 55, 1205. https://doi.org/10.1364/JOSA.55.001205.
112. Saleh, B.E.A.; Teich, M.C. Fundamentals of Photonics; 1st ed.; Wiley: Hoboken, NJ, USA, **1991**; ISBN 978-0-471-83965-1.
113. Topasna, D.M.; Topasna, G.A. Numerical Modeling of Thin Film Optical Filters. In Proceedings of the Education and Training in Optics and Photonics; OSA: St. Asaph, North Wales, **2009**; p. EP5.
114. Kajikawa, K.; Okamoto, T. Optical Electromagnetic Field Analysis Using Python: Practical Application in Metallic and Dielectric Nanostructures; 1st ed.; CRC Press: Boca Raton, FL, USA, **2025**; ISBN 978-1-003-35767-4.
115. Byrnes, S.J. Multilayer Optical Calculations *arXiv* **2016**, arXiv:1603.02720. https://doi.org/10.48550/arXiv.1603.02720.

116. Dovzhenko, D.; Martynov, I.; Samokhvalov, P.; Osipov, E.; Lednev, M.; Chistyakov, A.; Karaulov, A.; Nabiev, I. Enhancement of Spontaneous Emission of Semiconductor Quantum Dots inside One-Dimensional Porous Silicon Photonic Crystals. Opt. Express **2020**, 28, 22705. https://doi.org/10.1364/OE.401197.
117. Granizo, E.; Kriukova, I.; Escudero-Villa, P.; Samokhvalov, P.; Nabiev, I. Enhanced Fluorescence Emission of a Single Quantum Dot in a Porous Silicon Photonic Crystal—Plasmonic Hybrid Resonator. J. Phys.: Conf. Ser. **2024**, 2796, 012021. https://doi.org/10.1088/1742-6596/2796/1/012021.
