# Supplementary material for: Enhanced Light–Matter Interaction in Porous Silicon Microcavities Structurally Optimized Using Theoretical Simulation and Experimental Validation
Source: Nanomaterials (Basel). 2025 Nov 29;15(23):1808. doi: 10.3390/nano15231808 (PMC12693180; doi:10.3390/nano15231808)
Supplement: Supplementary file 1 [file nanomaterials-15-01808-s001.zip › TMM and Bruggeman model_CORRECTED.pdf]

SECTION S7.
An example of calculation of the parameters and optical properties of a porous silicon microcavity using the transfer matrix model and Bruggeman approximation

The model is valid in the range of 369-1400 nm.
Silicon dispersion data is in the range 369-1400 nm.

$\lambda := 369 .. 1400$

Approximation of the real part of the complex refractive index of Si, based on published data:

$$nSical(\lambda) := 36036 \cdot e^{\left(\frac{-\lambda}{37.8}\right)} + 11.96 \cdot e^{\left(\frac{-\lambda}{152}\right)} + 0.892 \cdot e^{\left(\frac{-\lambda}{499}\right)} + 3.435$$

Alternatively, the real part of the complex refractive index of Si can be approximated by Sellmeier equation:

$$nSical2(\lambda) := \sqrt{1 + \frac{10.6684293 \cdot \lambda^2}{\lambda^2 - 301.516485^2} + \frac{0.0030434748 \cdot \lambda^2}{\lambda^2 - 1134.751153^2} + \frac{1.54133408 \cdot \lambda^2}{\lambda^2 - 1104000^2}}$$

Approximation of the imaginary part of the complex refractive index of Si, based on published data:

$$kSical(\lambda) := \begin{array}{|l} y \leftarrow 1.73658 \cdot 10^{13} \cdot \exp\left(\frac{-\lambda}{12.3403}\right) + 0.14788 \quad \text{if} \quad 380 \le \lambda < 420 \\ y \leftarrow 6855.42451 \cdot \exp\left(\frac{-\lambda}{38.90007}\right) + 0.02607 \quad \text{if} \quad 420 \le \lambda < 520 \\ y \leftarrow 0.96502 \cdot \exp\left(\frac{-\lambda}{156.96774}\right) - 7.39905 \cdot 10^{-4} \quad \text{if} \quad 520 \le \lambda < 1000 \\ 0 \quad \text{otherwise} \end{array}$$

Approximation of the real and imaginary part of the complex refractive index of SiO2:

$$nSiO2cal(\lambda) := \sqrt{1 + \frac{0.6961663 \cdot \lambda^2}{\lambda^2 - (68.4043)^2} + \frac{0.4079426 \cdot \lambda^2}{\lambda^2 - 116.2414^2} + \frac{0.8974794 \cdot \lambda^2}{\lambda^2 - (9896.161)^2}}$$

$$kSiO2cal(\lambda) := 0$$

Total complex refractive indices of Si and SiO2:

$$nTSical(\lambda) := nSical(\lambda) - i \cdot kSical(\lambda)$$

$$nTSiO2cal(\lambda) := nSiO2cal(\lambda) - i \cdot kSiO2cal(\lambda)$$

General parameters:

$\rho := 0 .. 100$ — Porosity parameter

$\rho1 := 0 .. 100$ — SiO2 fraction in Si / SiO2 system

$n := 1$ — Refractive index of the pore medium (default: air)

$\lambda c \equiv 600$ — Target resonance wavelength of the microcavity

$nSi(\lambda) := nTSical(\lambda)$

$nSiO2(\lambda) := nTSiO2cal(\lambda)$

} Denotation of constants for further equations

$\varepsilon Si(\lambda) := nSi(\lambda)^2$ — Permittivity of Si

$\varepsilon SiO2(\lambda) := nSiO2(\lambda)^2$ — Permittivity of SiO2

Bruggeman model, 2-component system: Si / SiO2

$$A1(\rho1,\lambda) := \left(3\cdot\frac{\rho1}{100} - 1\right)\cdot(\varepsilon SiO2(\lambda) - \varepsilon Si(\lambda)) + \varepsilon Si(\lambda)$$

Intermediate variable:
-b in the quadratic equation $ax^2+bx+c = 0$,
with $x = \varepsilon_{SiO}$

$$\varepsilon Si\_SiO2(\rho1,\lambda) := \frac{A1(\rho1,\lambda) + \sqrt{(A1(\rho1,\lambda))^2 + 8\,\varepsilon Si(\lambda)\cdot\varepsilon SiO2(\lambda)}}{4}$$

Permittivity of 2-component system: Si / SiO2

$$nSiO(\rho1,\lambda) := \sqrt{\varepsilon Si\_SiO2(\rho1,\lambda)}$$

Effective refractive index of Si / SiO2 system

Bruggeman model, 3-component system: Air / Si / SiO2

$$A2(\rho,\rho1,\lambda) := \left(3\cdot\frac{\rho}{100} - 1\right)\cdot\left(n^2 - \varepsilon Si\_SiO2(\rho1,\lambda)\right) + \varepsilon Si\_SiO2(\rho1,\lambda)$$

Intermediate variable:
-b in the quadratic equation $ax^2+bx+c = 0$,
with $x = \varepsilon_{eff}$

$$\varepsilon effBr(\rho,\rho1,\lambda) := \frac{A2(\rho,\rho1,\lambda) + \sqrt{(A2(\rho,\rho1,\lambda))^2 + 8\,\varepsilon Si\_SiO2(\rho1,\lambda)\cdot n^2}}{4}$$

Permittivity of Air / Si / SiO2 system

$$neffBr(\rho,\rho1,\lambda) := \sqrt{\varepsilon effBr(\rho,\rho1,\lambda)}$$

Effective refractive index of porous silicon

Approximation of calibration curves (I [mA]):

$v(I) := 1.534 + 0.709\cdot I$

$p(I) := 52.48 + 0.942\cdot I$

Let's consider non-oxidized sample: $\rho1 := 0$

	High porosity layer	Low porosity layer	Si substrate
Etching current I, mA:	$IH := 26.8$	$IL := 3.5$	
Etching rate, nm/s:	$vH := v(IH) = 20.535$	$vL := v(IL) = 4.016$	
Porosity, %:	$pH := p(IH) = 77.726$	$pL := p(IL) = 55.777$	
Effective refractive indices according to Bruggeman model:	$nH(\lambda) := neffBr(pH, \rho1, \lambda)$	$nL(\lambda) := neffBr(pL, \rho1, \lambda)$	
	$nH(\lambda c) = 1.411 - 0.002i$	$nL(\lambda c) = 2.137 - 0.007i$	$nSi(\lambda c) = 3.939 - 0.02i$
	$Re(nH(\lambda c)) = 1.411$	$Re(nL(\lambda c)) = 2.137$	
Required layer thickness, nm:	$dH := \frac{\lambda c}{4 \cdot Re(nH(\lambda c))} = 106.33$	$dL := \frac{\lambda c}{4 \cdot Re(nL(\lambda c))} = 70.18$	$dSi := 500$
Required etching time, s:	$tH := \frac{dH}{vH} = 5.178$	$tL := \frac{dL}{vL} = 17.477$	
Phase in the homogeneous layer:	$\varphi H(\lambda) := nH(\lambda) \cdot dH \cdot \frac{2\pi}{\lambda}$	$\varphi L(\lambda) := nL(\lambda) \cdot dL \cdot \frac{2\pi}{\lambda}$	$\varphi Si(\lambda) := nL(\lambda) \cdot dL \cdot \frac{2\pi}{\lambda}$

Transfer matrices for all layer ***interfaces***:

air / nL

$$M0(\lambda) := \begin{bmatrix} \frac{(nL(\lambda) + n)}{2 \cdot nL(\lambda)} & \frac{(nL(\lambda) - n)}{2 \cdot nL(\lambda)} \\ \frac{(nL(\lambda) - n)}{2 \cdot nL(\lambda)} & \frac{(nL(\lambda) + n)}{2 \cdot nL(\lambda)} \end{bmatrix}$$

nH / nL

$$M1(\lambda) := \begin{bmatrix} \frac{(nL(\lambda) + nH(\lambda))}{2 \cdot nL(\lambda)} & \frac{(nL(\lambda) - nH(\lambda))}{2 \cdot nL(\lambda)} \\ \frac{(nL(\lambda) - nH(\lambda))}{2 \cdot nL(\lambda)} & \frac{(nL(\lambda) + nH(\lambda))}{2 \cdot nL(\lambda)} \end{bmatrix}$$

nL / air

$$M2(\lambda) := \begin{bmatrix} \frac{(n + nL(\lambda))}{2 \cdot n} & \frac{(n - nL(\lambda))}{2 \cdot n} \\ \frac{(n - nL(\lambda))}{2 \cdot n} & \frac{(n + nL(\lambda))}{2 \cdot n} \end{bmatrix}$$

nL / nH

$$M3(\lambda) := \begin{bmatrix} \frac{(nH(\lambda) + nL(\lambda))}{2 \cdot nH(\lambda)} & \frac{(nH(\lambda) - nL(\lambda))}{2 \cdot nH(\lambda)} \\ \frac{(nH(\lambda) - nL(\lambda))}{2 \cdot nH(\lambda)} & \frac{(nH(\lambda) + nL(\lambda))}{2 \cdot nH(\lambda)} \end{bmatrix}$$

nL / substrate

$$M4(\lambda) := \begin{bmatrix} \frac{(nSi(\lambda) + nL(\lambda))}{2 \cdot nSi(\lambda)} & \frac{(nSi(\lambda) - nL(\lambda))}{2 \cdot nSi(\lambda)} \\ \frac{(nSi(\lambda) - nL(\lambda))}{2 \cdot nSi(\lambda)} & \frac{(nSi(\lambda) + nL(\lambda))}{2 \cdot nSi(\lambda)} \end{bmatrix}$$

Transfer matrices for all ***homogeneous layers***:

High porosity layer

$$MH(\lambda) := \begin{pmatrix} e^{-i \cdot \varphi H(\lambda)} & 0 \\ 0 & e^{i \cdot \varphi H(\lambda)} \end{pmatrix}$$

Low porosity layer

$$ML(\lambda) := \begin{pmatrix} e^{-i \cdot \varphi L(\lambda)} & 0 \\ 0 & e^{i \cdot \varphi L(\lambda)} \end{pmatrix}$$

Si substrate

$$MSi(\lambda) := \begin{pmatrix} e^{-i \cdot \varphi Si(\lambda)} & 0 \\ 0 & e^{i \cdot \varphi Si(\lambda)} \end{pmatrix}$$

Number of layer pairs in the structure $(LH)_{N1}L_2(LH)_{N2}$

$N1 := 4 \qquad N2 := 5$

Total matrix of the system: matrices are multiplied in the reverse order of the system topology.
The matrix for the segment closest to the system's input is the last to multiply on the left

$$M(\lambda) := MSi(\lambda)\cdot(M4(\lambda)\cdot ML(\lambda)\cdot M1(\lambda)\cdot MH(\lambda))\cdot(M3(\lambda)\cdot ML(\lambda)\cdot M1(\lambda)\cdot MH(\lambda))^{N2-1}\cdot\ldots$$

$$\ldots\cdot M3(\lambda)\cdot ML(\lambda)^{2}\cdot(M1(\lambda)\cdot MH(\lambda)\cdot M3(\lambda)\cdot ML(\lambda))^{N1-1}\cdot M1(\lambda)\cdot(MH(\lambda)\cdot M3(\lambda)\cdot ML(\lambda)\cdot M0(\lambda))$$

$$Refl(\lambda) := 100\left(\left|\frac{M(\lambda)_{2,1}}{M(\lambda)_{2,2}}\right|\right)^2$$

$$Transm(\lambda) := 100\cdot\frac{Re(nSi(\lambda))}{n}\left(\left|\frac{M(\lambda)_{1,1}\cdot M(\lambda)_{2,2} - M(\lambda)_{1,2}\cdot M(\lambda)_{2,1}}{M(\lambda)_{2,2}}\right|\right)^2$$

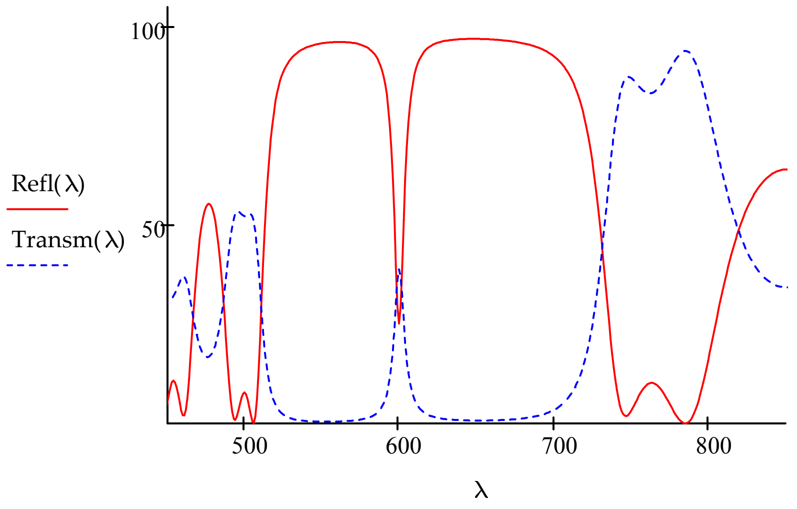

λ =	Refl(λ) =
369	66.002
370	55.453
371	45.88
372	51.839
373	77.061
374	90.883
375	95.761
376	97.603
377	98.389
378	98.744
379	98.885
380	29.689
381	28.409
382	26.827
383	24.952
...	...
